# Supplementary material for: STIC2 selectively binds ribosome-nascent chain complexes in the cotranslational sorting of Arabidopsis thylakoid proteins
Source: EMBO J. 2024 Aug 27;43(20):4699–719. doi: 10.1038/s44318-024-00211-4 (PMC11480477; doi:10.1038/s44318-024-00211-4)
Supplement: Supplementary file 1 — Appendix [file 44318_2024_211_MOESM1_ESM.pdf]

Appendix for

STIC2 selectively binds ribosome-nascent chain complexes in the cotranslational sorting of *Arabidopsis* thylakoid proteins

Stolle, Osterhoff et al.

Content:

Appendix Figures:

Appendix Figure S1 Sucrose gradient enrichment and affinity purification of ribosomes translating TST-tagged peptides..p2
Appendix Figure S2 Comparison of proteins identified in TST-D1 and TST-uS2c RNCs...p3
Appendix Figure S3 Verification of homozygous *ffc stic2* plants.......................................p4
Appendix Figure S4 Characterization of *Arabidopsis* WT and mutant plants.....................p5
Appendix Figure S5 Membrane bound Alb3- and Alb4-peptide arrays used for the interaction analyses with STIC2..p6
Appendix Figure S6 The binding interface of STIC2 with Alb4C and Alb3C.......................p7

Appendix Tables:

Appendix Table S1 Primer...p8-9

Appendix Supplementary Materials and Methods..p10

Appendix Supplementary References...p11

Appendix Figures

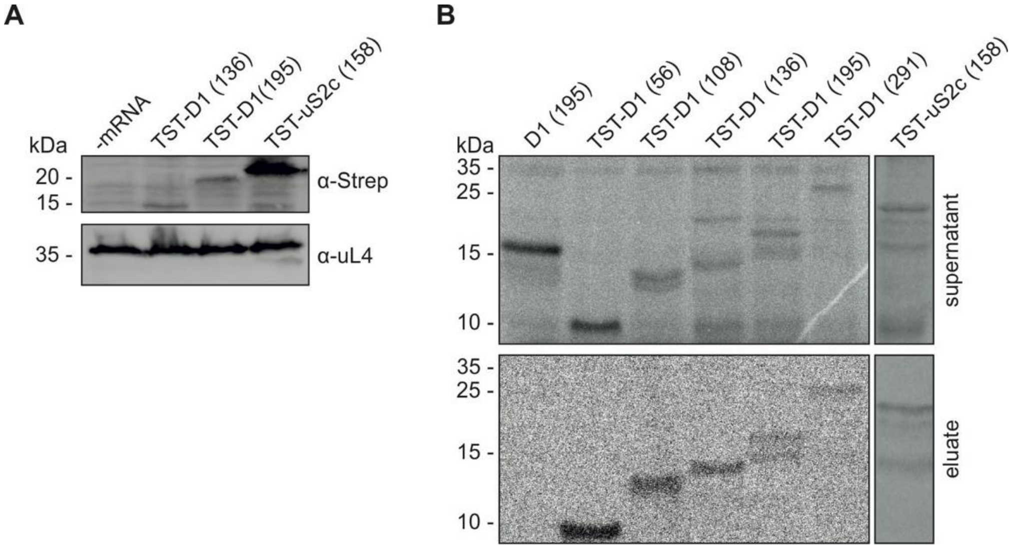

Appendix Figure S1 Sucrose gradient enrichment and affinity purification of ribosomes translating TST-tagged peptides

Stalled RNCs with nascent TST-D1 peptides of the indicated lengths and TST-uS2c (158) were generated using the pea chloroplast-derived *in vitro* translation system. Details on the various constructs are given in Figure 1.

A. Enrichment of TST-D1 (136), TST-D1 (195), and TST-uS2c (158) RNCs via 1 M sucrose cushion centrifugation. A translation sample lacking mRNA was used as a control. The sedimented protein complexes were separated with SDS-PAGE and applied to Western blot analysis. RNCs with 15 kDa and 20 kDa TST-D1 or 22 kDa TST-uS2c peptides were detected with antibody against the TST (α-Strep) and the ribosomal protein uL4 (α-uL4).

B. In the presence of radiolabeled methionine, truncated D1 and uS2c peptides were synthesized *in vitro* and subsequently incubated with MagStrep XT magnetic beads for affinity-based purification. A truncated D1 (195) peptide lacking the TST was synthesized as a negative control for the affinity purification (first lane). Unbound RNCs were removed with the supernatant in a magnetic separator. The magnetic beads were washed and remaining RNCs were subsequently released with native elution. Supernatant (upper panel) and eluate (lower panel) were subjected to SDS-PAGE and synthesized peptides were detected by phosphor imaging.

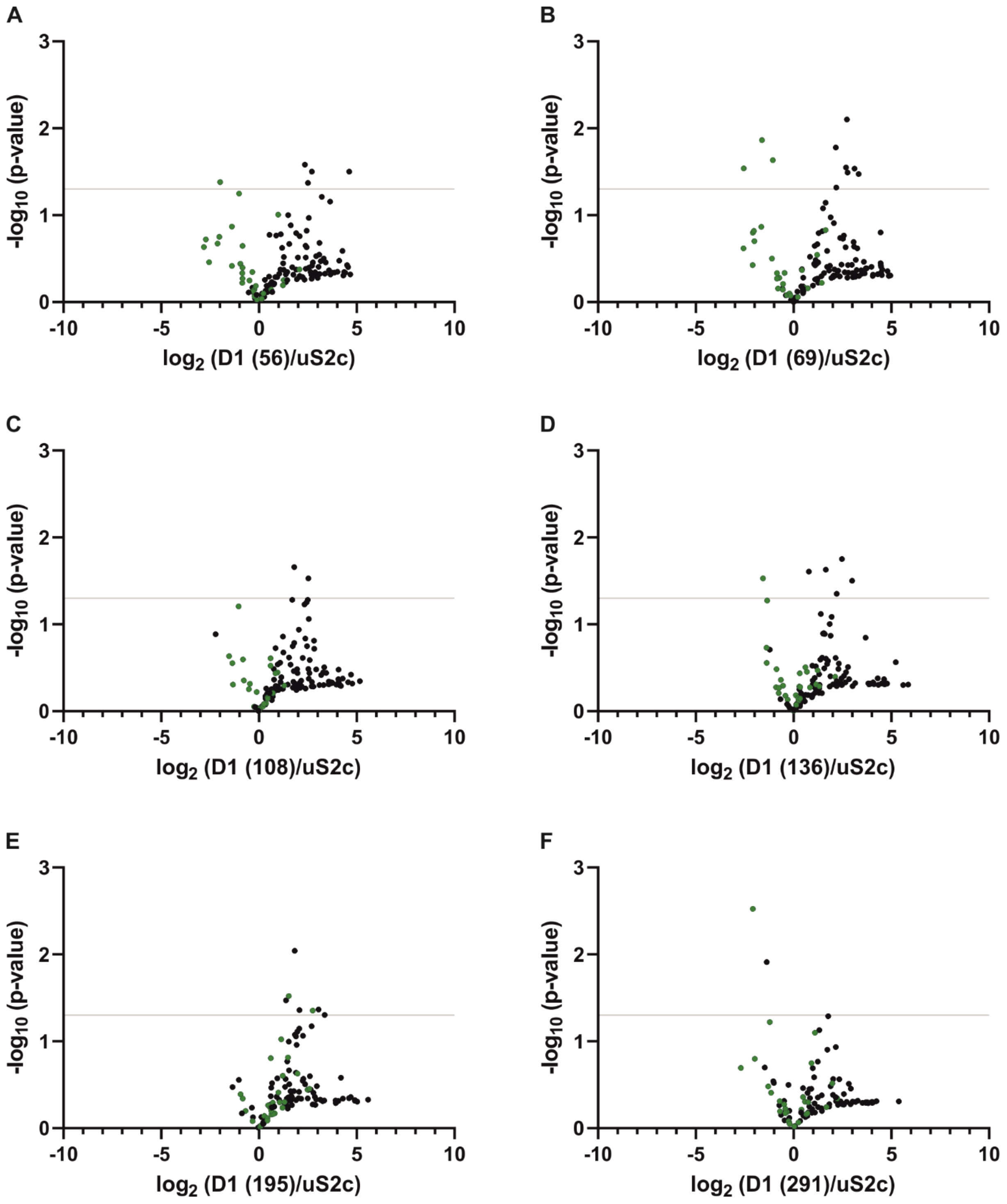

Appendix Figure S2 Comparison of proteins identified in TST-D1 and TST-uS2c RNCs

A-**F**. Volcano plots representing proteins identified by tandem MS in RNCs with TST-uS2c (158) and TST-D1 peptides of different length. *P*-values (-$\log_{10}$) were plotted against the ratio of label-free quantification intensity means ($\log_2$). The *p*-values were determined by two-sided *t*-test ($p \leq 0.05$). Ribosomal proteins are labeled in green.

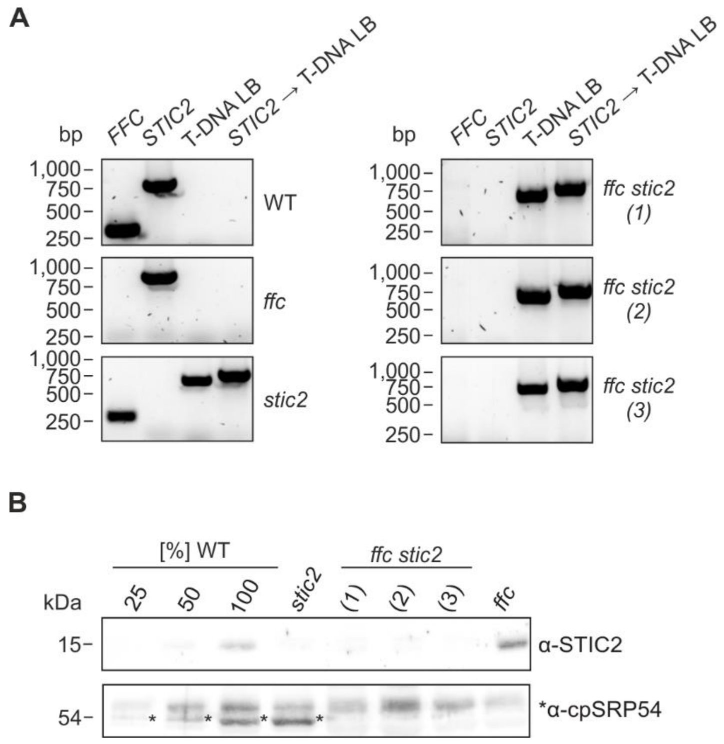

Appendix Figure S3 Verification of homozygous *ffc stic2* plants

The *Arabidopsis thaliana* double mutant *ffc1-2 stic2-3* (*ffc stic2*) was created by crossing the homozygous single mutant plants *ffc1-2* (*ffc*) and *stic2-3* (*stic2*) with *stic2* as female plant. Putative *ffc stic2* double mutants (*ffc stic2* (1), (2), and (3)) of the F2 generation were verified via genotyping PCRs (**A**) and immunoblotting (**B**).

A. Wild-type plants of the ecotype Columbia-0 (Col-0), the *ffc* and *stic2* mutants were used as controls. Gene-specific primer combinations were used to prove DNA insertions in ffc (At5g03940, lane FFC) and stic2 (At2g24020, lane STIC2). The T-DNA insertion was verified by amplifying its left border (T-DNA LB). Proof of proper T-DNA insertion in stic2 was analyzed by amplification of the junction from the 5’UTR to the T-DNA left border. Bands at 319, 821, about 600 and 750 bp display PCR products of ffc, stic2, the T-DNA left border and the junction from stic2 5’ UTR to the T-DNA left border.

B. Total protein extracts from leaves of 5-week-old *Arabidopsis thaliana* plants (wild-type (Col-0), *stic2, ffc* and the putative double mutants *ffc stic2 (1), (2) and (3)* were separated by SDS-PAGE and analyzed via immunoblotting regarding the expression of cpSRP54 and STIC2. The loading control is the same as in Figure 3.

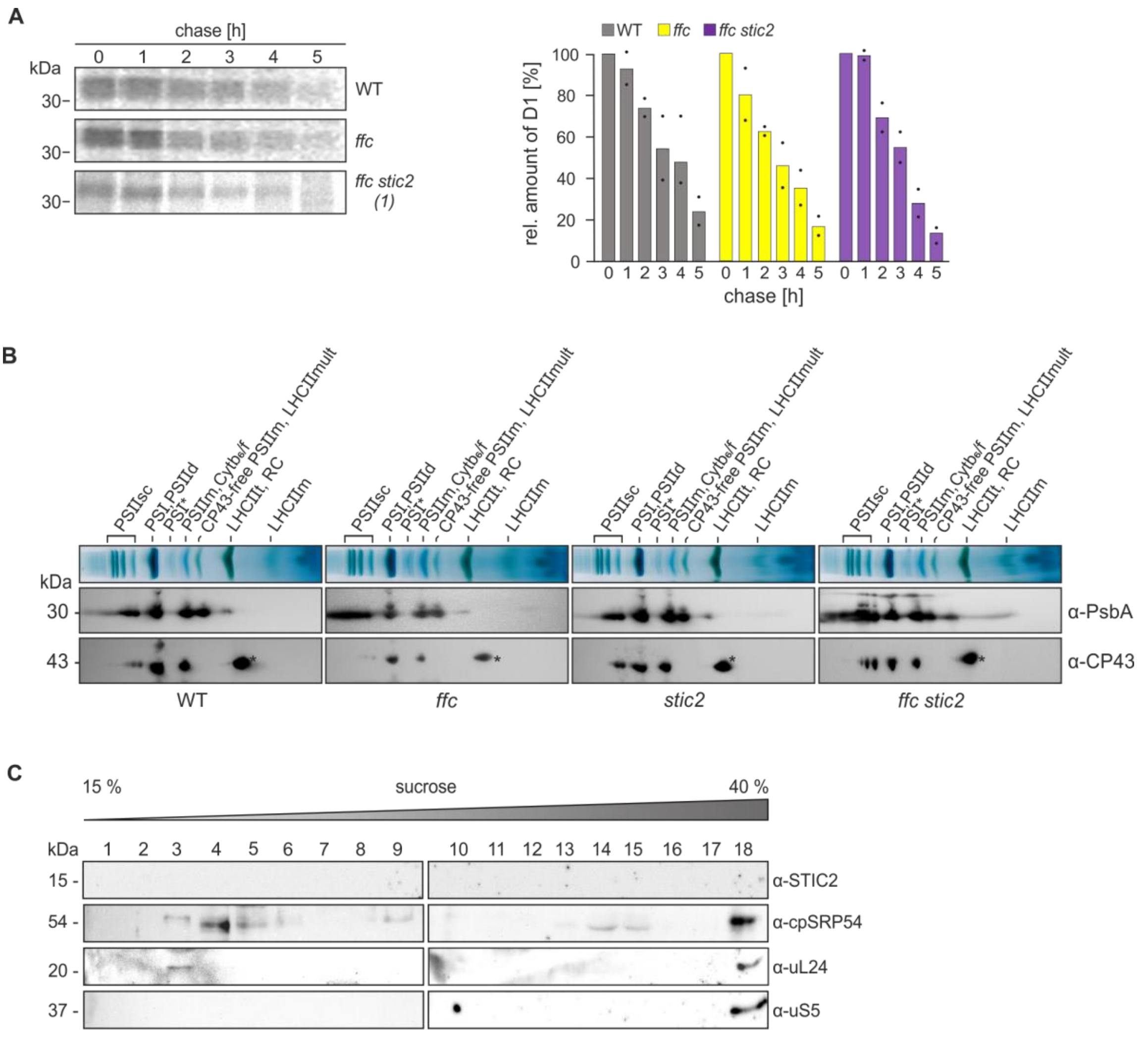

Appendix Figure S4 Characterization of *Arabidopsis* WT and mutant plants

A. Leaf discs of wild type (Col-0) and the indicated *A. thaliana* mutants were labeled with [^{35}S]-methionine in presence of cycloheximide for 60 min in ambient light (0 h chase). After removal of [^{35}S]-methionine the labeled D1 protein was chased for 1, 2, 3, 4 and 5 hours under high light (1000 µmol photons m^{-2} s^{-1}). Labeled thylakoid membrane proteins were used for SDS-PAGE and phosphor imaging. Signals from two independent experiments were quantified in relation to 0 h chase time using ImageJ.

B. Thylakoids isolated from chloroplasts of *Arabidopsis thaliana* plants (Col-0, *ffc1-2* (*ffc*), *stic2-3* (*stic2*) and *ffc1-2 stic2-3* (*ffc stic2*)) were solubilized in DDM and separated by BN-PAGE based on equal chlorophyll loading (10 µg chlorophyll/lane). The identification of detected bands was accomplished in accordance with published BN-PAGE profiles of Arabidopsis thylakoids (Granvogl et al. 2006; Armbruster et al. 2010; Wittenberg et al. 2017; Che et al. 2022). Photosystem I and II (PSI and II), PSII supercomplexes ($PSII_{sc}$), PSII dimers ($PSII_d$), PSI assembly intermediate (PSI*), monomeric PSII ($PSII_m$) and Cytochrom b_6/f complex ($Cytb_6$/f), CP43-free PSII monomers (CP43-free $PSII_m$) and multimeric light harvesting antenna complex II ($LHCII_{mult}$), trimeric LHCII ($LHCII_t$), reaction center-like complex (RC), monomeric ($LHCII_m$). Additionally, the protein complex subunit composition was determined by two-dimensional BN-PAGE/SDS-PAGE analysis followed by immunoblot analysis using α-PsbA and α-CP43 antibodies (Agrisera) directed against the PS II subunits D1 and CP43, respectively. The asterisk (*) marks free CP43 protein.

C. A cell extract of the *stic2-3* plant was subjected to sucrose gradient centrifugation in the presence of chloramphenicol. Gradient fractions were separated by SDS-PAGE and applied to immunoblotting using the indicated antibodies.

A

Alb3 155-208

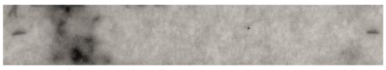

1 TYPLTKQQVESTLAM
2 TKQQVESTLAMQNLQ
3 VESTLAMQNLQPKIK
4 LAMQNLQPKIKAIQQ
5 NLQPKIKAIQQRYAG
6 KIKAIQQRYAGNQER
7 IQQRYAGNQERIQLE
8 YAGNQERIQLETSRL
9 QERIQLETSRLYKQA
10 QLETSRLYKQAGVNP
11 TSRLYKQAGVNPLAG

Alb3 282-462

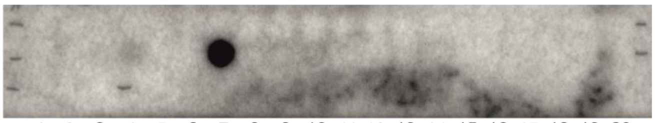

1 LVLPVLLIASQYVSM
2 VLLIASQYVSMEIMK
3 ASQYVSMEIMKPPQT
4 VSMEIMKPPQTDDPA
5 IMKPPQTDDPAQKNT
6 PQTDDPAQKNTLLVF
7 DPAQKNTLLVFKFLP
8 KNTLLVFKFLPLMIG
9 LVFKFLPLMIGYFAL
10 FLPLMIGYFALSVPS
11 MIGYFALSVPSGLSI
12 FALSVPSGLSIYWLT
13 VPSGLSIYWLTNNVL
14 LSIYWLTNNVLSTAQ
15 WLTNNVLSTAQQVYL
16 NVLSTAQQVYLRKLG
17 TAQQVYLRKLGGAKP
18 VYLRKLGGAKPNMDE
19 KLGGAKPNMDENASK
20 AKPNMDENASKIISA
21 MDENASKIISAGRAK
22 ASKIISAGRAKRSIA
23 ISAGRAKRSIAQPDD
24 RAKRSIAQPDDAGER
25 SIAQPDDAGERFRQL
26 PDDAGERFRQLKEQE
27 GERFRQLKEQEKRSK
28 RQLKEQEKRSKKNKA
29 EQEKRSKKNKAVAKD
30 RSKKNKAVAKDTVEL
31 NKAVAKDTVELVEES
32 AKDTVELVEESQSES
33 VELVEESQSESEEGS
34 EESQSESEEGSDDEE
35 SESEEGSDDEEEEAR
36 EGSDDEEEEAREGAL
37 DEEEEAREGALASST
38 EAREGALASSTTSKP
39 GALASSTTSKPLPEV
40 SSTTSKPLPEVGQRR
41 SKPLPEVGQRRSKRS
42 PEVGQRRSKRSKRKR
43 VGQRRSKRSKRKRTV

B

Alb4 139-192

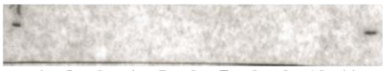

1 TFPLTKKQVESAMAM
2 TKKQVESAMAMKSLT
3 VESAMAMKSLTPQIK
4 MAMKSLTPQIKAIQE
5 SLTPQIKAIQERYAG
6 QIKAIQERYAGDQEK
7 IQERYAGDQEKIQLE
8 YAGDQEKIQLETARL
9 QEKIQLETARLYKLA
10 QLETARLYKLAGINP
11 TARLYKLAGINPLAG

Alb4 266-499

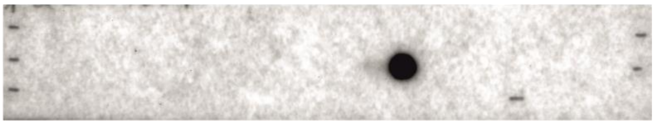

1 LVLPLLLVFSQYLSI
2 LLLVFSQYLSIQIMQ
3 FSQYLSIQIMQSSQS
4 LSIQIMQSSQSNDPA
5 IMQSSQSNDPAMKSS
6 SQSNDPAMKSSQAVT
7 DPAMKSSQAVTKLLP
8 KSSQAVTKLLPLMIG
9 AVTKLLPLMIGYFAL
10 LLPLMIGYFALSVPS
11 MIGYFALSVPSGLSL
12 FALSVPSGLSLYWLT
13 VPSGLSLYWLTNNIL
14 LSLYWLTNNILSTAQ
15 WLTNNILSTAQQVWL
16 NILSTAQQVWLQKYG
17 TAQQVWLQKYGGAKN
18 VWLQKYGGAKNPVEK
19 KYGGAKNPVEKFTNL
20 AKNPVEKFTNLVTKE
21 VEKFTNLVTKEDKTQ
22 TNLVTKEDKTQQIEK
23 TKEDKTQQIEKSFSE
24 KTQQIEKSFSEPLVQ
25 IEKSFSEPLVQKSVS
26 FSEPLVQKSVSELKI
27 LVQKSVSELKIPREK
28 SVSELKIPREKGGEK
29 LKIPREKGGEKVTPE
30 REKGGEKVTPESPKP
31 GEKVTPESPKPGERF
32 TPESPKPGERFRLLK
33 PKPGERFRLLKEQEA
34 ERFRLLKEQEAKRRR
35 LLKEQEAKRRREKEE
36 QEAKRRREKEERQKA
37 RRREKEERQKAEAAL
38 KEERQKAEAALSNQN
39 QKAEAALSNQNTDKA
40 AALSNQNTDKAHEQD
41 NQNTDKAHEQDEKSD
42 DKAHEQDEKSDTAIV
43 EQDEKSDTAIVAEDD
44 KSDTAIVAEDDKKTE
45 AIVAEDDKKTELSAV
46 EDDKKTELSAVDETS
47 KTELSAVDETSDGTV
48 SAVDETSDGTVAVNG
49 ETSDGTVAVNGKPSI
50 GTVAVNGKPSIQKDE
51 VNGKPSIQKDETTNG
52 PSIQKDETTNGTFGI
53 KDETTNGTFGIGHDT
54 TNGTFGIGHDTEQQH
55 FGIGHDTEQQHSHET
56 GHDTEQQHSHETEKR

Appendix Figure S5 Membrane bound Alb3- and Alb4-peptide arrays used for the interaction analyses with STIC2

A. Peptides covering Alb3 residues 155-208 and 282-462 (15mer peptides) were incubated with His-mSTIC2 (aa 49-182) at a final concentration of 5 µg/ml. The peptide sequences are indicated below the pepspot membranes. An antibody-HRP conjugate against the His-tag was used to detect His-mSTIC2 protein on pepspot membranes.

B. Peptides covering Alb4 residues 139-192 and 266-499 were analyzed as in **A**.

Further details are given in Materials and Methods and Figure 5A in the main text. The peptide arrays shown in the right panels of this figure and in Figure 5A are identical.

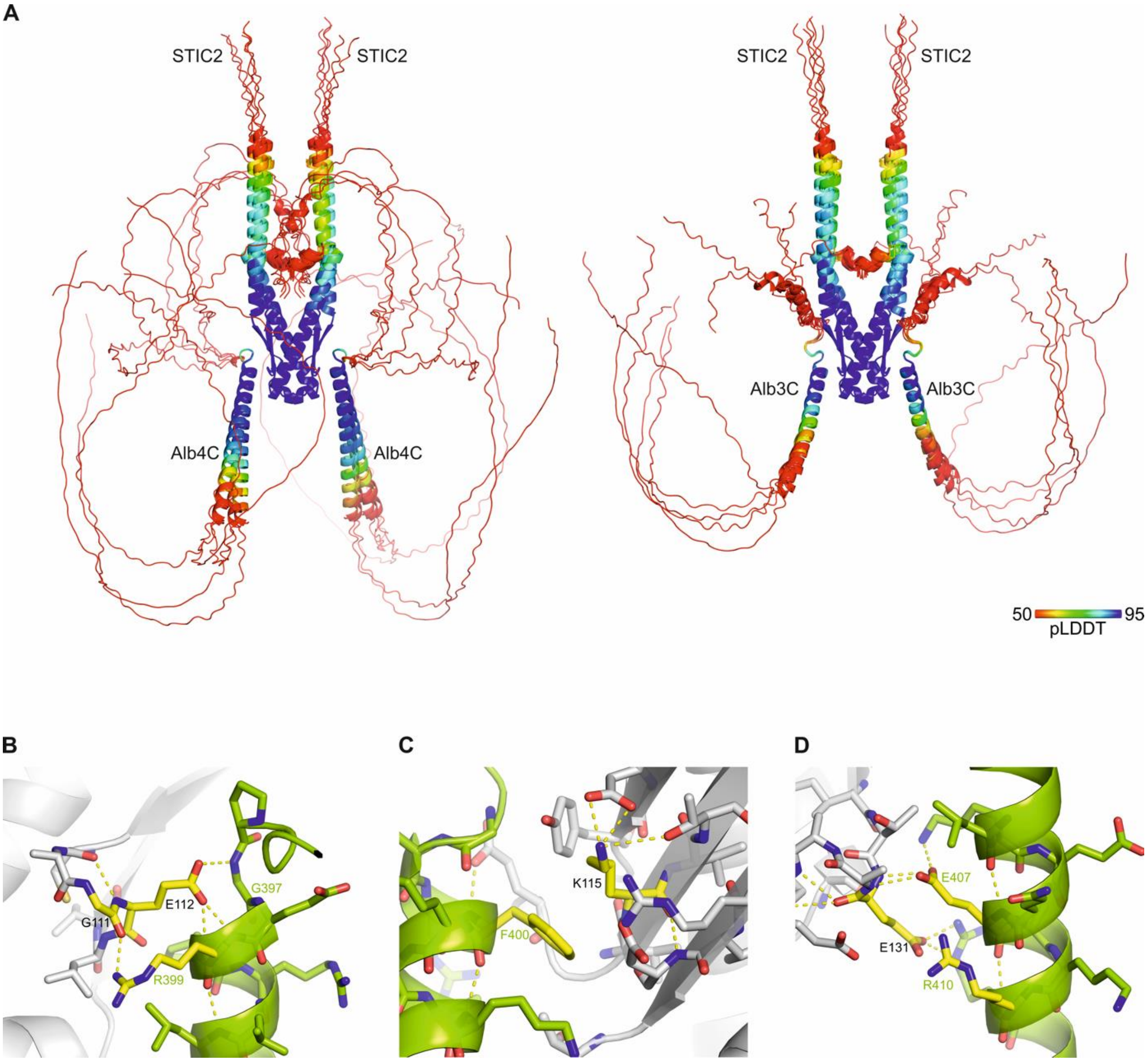

Appendix Figure S6 The binding interface of STIC2 with Alb4C and Alb3C

A. Alphafold-Multimer models of STIC2 in complex with Alb4C (left) and Alb3C (right). The five models of each prediction run are superposed in cartoon representation. Coloring according to the residue specific pLDDT quality score from 50-95 (see inset), blue being the most reliable.

B-D. Interactions between STIC2 and Alb4C. Detailed view of interactions of residues discussed in the text. Proteins are shown in cartoon representation in grey and green for STIC2 and Alb4C, respectively. Residues under investigation are shown as yellow sticks with their polar interactions as defined in Pymol. In addition, residues within 4 Å are shown as grey/green sticks.

Appendix Tables

Appendix Table S1 Primer

Construct	Sequence (5' → 3')	Cloning strategy
**Generation of DNA templates for *in vitro* transcription**		
TST-D1 (aa 1-56)	for: GTAATACGACTCACTATAGGGCGAGTAACAAGC CCTTAATTCTATAGTTA<br>rev: AGGGGCAGCAATGAAAGCG	
TST-D1 (aa 1-69)	for: GTAATACGACTCACTATAGGGCGAGTAACAAGC CCTTAATTCTATAGTTA<br>rev: TCCAGAAACAGGCTCACGAATACC	
TST-D1 (aa 1-108)	for: GTAATACGACTCACTATAGGGCGAGTAACAAGC CCTTAATTCTATAGTTA<br>rev: CGTTGTATAACCATTCATCAACGGATGC	
TST-D1 (aa 1-136)	for: GTAATACGACTCACTATAGGGCGAGTAACAAGC CCTTAATTCTATAGTTA<br>rev: GACGAAAACTAAGTTCCCACTCACG	
TST-D1 (aa 1-195)	for: GTAATACGACTCACTATAGGGCGAGTAACAAGC CCTTAATTCTATAGTTA<br>rev: GGTGCATAAGAATATTATGCTCAGCC	
TST-D1 (aa 1-291)	for: GTAATACGACTCACTATAGGGCGAGTAACAAGC CCTTAATTCTATAGTTA<br>rev: GCTGATACCTAACGCGGTAAACCAG	
TST-uS2c (aa 1-58)	for: TAATACGACTCACTATAGGG<br>rev: CATATATTTAATCCCGCCTAGATATG	
**Genotyping PCRs**		
FFC	for: GATAAAGGCATGATGGACGAATTAAAAGACG<br>rev: CAAACCTCCTTGACACTCAAAGCAGCACCACC	
STIC2	for: CAATCTAAGCTCAAAAAAAGAGTTAAAGTGACG<br>rev: GAACACGTACAGCTTCCACTTGAACAACC	
T-DNA LB	for: CTGCCTGTATCGAGTGGTGA<br>rev: ACTTAATCGCCTTGCAGCAC	
STIC2 → T-DNA LB	for: CAATCTAAGCTCAAAAAAAGAGTTAAAGTGACG<br>rev: ACTTAATCGCCTTGCAGCAC	
**pET-Duet1-**		
STIC2 (aa 49-182)	for: CATCACCATCATCACCACAGCCAGGTGAATGGA TTATTTGGAGGTGGAA<br>rev: GCGGCCGCAAGCTTGTCGACTTACTTCATTCC TTCGCTGA	InFusion
STIC2 (aa 64-182)	for: TCATCACCACAGCCAGGATCCGGATGGACAAT CAAAGGCAGGA<br>rev: GCGGCCGCAAGCTTGTCGACTTACTTCATTCC TTCGCTGA	InFusion
STIC2 G111L	for: GATGGTTATTGTGCATTAGAGCTTGTCAAGGTT AC<br>rev: GTAACCTTGACAAGCTCTAATGCACAATAACCA TC	QuikChange mutagenesis

STIC2 E112A	for: GGTTATTGTGCAGGCGCGCTTGTCAAGGTTACG<br>rev: CGTAACCTTGACAAGCGCGCCTGCACAATAACC	QuikChange mutagenesis
STIC2 K115A	for: GCAGGCGAGCTTGTCGCGGTTACGTTATCAGG<br>rev: CCTGATAACGTAACCGCGACAAGCTCGCCTGC	QuikChange mutagenesis
STIC2 E131G	for: [5' phos] GGAGCAGCAATGGAACTAGGTTCC<br>rev: [5' phos] GGTAATATCAGTACGGATTGG	Site-directed mutagenesis
**pET29b-**		
Alb3CΔIII (aa 350-462 Δ386-403)	for: GGATGATGCGGCGGTGGCGAAAGATACCGT<br>rev: TCGCCACCGCCGCATCATCCGGCTGCGC	InFusion
Alb4CΔIII (aa 334-499 Δ397-414)	for: GAATGCCCCAAACCTGAAGAGAGGCAGAAAGCTGAAGCAG<br>rev: TTTCTGCCTCTCTTCAGGTTTGGGGCATTCTGGGGTCACC	InFusion
Alb4C R399G	for: [5'phos] GGGTTTAGGCTGCTGAAAGAGCAA<br>rev: [5'phos] TTCACCAGGTTTGGGGCATTC	Site-directed mutagenesis
Alb4C F400G	for: [5'phos] GGTAGGCTGCTGAAAGAGCAAGAA<br>rev: [5'phos] CCTTTCACCAGGTTTGGGGCA	Site-directed mutagenesis
Alb4C E407G	for: [5'phos] GGAGCAAAGAGACGTCGAGAAAAA<br>rev: [5'phos] TTGCTCTTTCAGCAGCCTAAA	Site-directed mutagenesis
Alb4C R410G	for: [5'phos] GGACGTCGAGAAAAAGAAGAGAGG<br>rev: [5'phos] CTTTGCTTCTTGCTCTTTCAGCAG	Site-directed mutagenesis
**pGEX4T3-**		
STIC2 (aa 49-182)	for: ATCTGGTTCCGCGTGGATCCGTGAATGGATTATTTGG<br>rev: GGCCGCTCGAGTCGACTTACTTCATTCCTTCGCTGA	InFusion
STIC2 G111L	for: ATCTGGTTCCGCGTGGATCCGTGAATGGATTATTTGG<br>rev: GGCCGCTCGAGTCGACTTACTTCATTCCTTCGCTGA	Classical cloning
STIC2 E112A	for: ATCTGGTTCCGCGTGGATCCGTGAATGGATTATTTGG<br>rev: GGCCGCTCGAGTCGACTTACTTCATTCCTTCGCTGA	Classical cloning
STIC2 K115A	for: ATCTGGTTCCGCGTGGATCCGTGAATGGATTATTTGG<br>rev: GGCCGCTCGAGTCGACTTACTTCATTCCTTCGCTGA	Classical cloning
STIC2 E112A K115A	for: [5'phos]GCGCTTGTCGCGGTTACGTTATCA<br>rev: [5'phos]GCCTGCACAATAACCATCAAATTC	Site-directed mutagenesis
STIC2 E131G	for: AGTGGATCCGATGGACAATCAAAGGCAGGA<br>rev: GGCCGCTCGAGTCGACTTACTTCATTCCTTCGCTGA	Classical cloning

Appendix Supplementary Materials and Methods

Sucrose gradient enrichment of RNCs

For sucrose cushion centrifugation 160 µl of the translation reaction were loaded on a 2.9 ml sucrose cushion (1 M sucrose, 30 mM HEPES pH 7.7, 9 mM magnesium acetate, 70 mM potassium acetate, 2 µg/ml antipain, 2 µg/ml leupeptin, 5 mM DTT, 0.25 mg/ml chloramphenicol, 0.1 mM AEBSF) and centrifuged (270,000 g, 90 min, 4°C, TLA 100.3, Beckman Coulter). The pellet was solubilized in sample buffer and subsequently applied to SDS-PAGE and Western blot analyses.

Affinity purification ^{35}S-methionine labelled RNCs

For SDS-PAGE and subsequent autoradiography analyses, the 40 µl *in vitro* translation reactions contained 20 µM 19 amino acid mix (Promega) and 0.45 µCi/µl of [^{35}S]-methionine (>1,000 Ci/mmol, Hartmann Analytics). The isolation of RNCs with MagStrep XT magnetic beads was essentially performed as described above with 30 µl of bead suspension.

Genomic DNA extraction and genotyping of Arabidopsis thaliana ffc stic2 double mutants

Potential *ffc stic2* double mutants were genotyped via PCR. Genomic DNA serving as template DNA was extracted according to Edwards et al. (1991) with the following modifications. After extraction of DNA from 2-3 small leaves, the air-dried DNA pellet was resuspended in 40 µl 10 mM Tris pH 6.7, 0.1 mM EDTA. Dissolved DNA is then incubated at 55°C for 15 min and briefly vortexed every 5 min. Subsequently, unresolved DNA and impurities were sedimented (5 min, 14,000 rpm, RT). The DNA-containing supernatant was further used for genotyping PCRs using GoTaq® Polymerase (Promega). The 10 kb DNA insertion into intron 8 in At5g03940 (*ffc*) was analyzed using a primer combination binding in exon 8 and 9 (Appendix Table S2). Primer binding in the 5' UTR of At2g24020 (*stic2*) and its coding sequence were used to analyze the T-DNA insertion into At2g24020. T-DNA insertion was verified by amplifying the T-DNA's left border and its localization within the 5' UTR of stic2 was verified by amplification of the transition from the stic2 5' UTR to the T-DNA.

Blue-native-polyacrylamide gel electrophoresis

Thylakoids isolated from chloroplasts of from *Arabidopsis thaliana* plants (Col-0, *ffc1-2* (*ffc*), *stic2-3* (*stic2*) and *ffc1-2 stic2-3* (*ffc stic2*)) were solubilized with 1.5% (w/v) n-dodecyl-β-D-maltosid (DDM) at a chlorophyll concentration of 1 mg/mL. The multiprotein complexes were separated by a 4-16% Blue-native-polyacrylamide gel electrophoresis (BN-PAGE) gradient according to the manufacturer's instructions (Invitrogen) using total amounts of 10 µg chlorophyll/lane.

Appendix Supplementary References

Armbruster U, Zühlke J, Rengstl B, Kreller R, Makarenko E, Rühle T, Schünemann D, Jahns P, Weisshaar B, Nickelsen J et al. (2010) The Arabidopsis thylakoid protein PAM68 is required for efficient D1 biogenesis and photosystem II assembly. *Plant Cell* 22: 3439–3460. DOI: 10.1105/tpc.110.077453.

Che L, Meng H, Ruan J, Peng L, Zhang L (2022) Rubredoxin 1 Is Required for Formation of the Functional Photosystem II Core Complex in Arabidopsis thaliana. *Front Plant Sci* 13: 824358. DOI: 10.3389/fpls.2022.824358.

Edwards K, Johnstone C, Thompson C (1991) A simple and rapid method for the preparation of plant genomic DNA for PCR analysis. *Nucleic Acids Res* 19: 1349. DOI: 10.1093/nar/19.6.1349

Granvogl B, Reisinger V, Eichacker LA (2006) Mapping the proteome of thylakoid membranes by de novo sequencing of intermembrane peptide domains. *Proteomics* 6: 3681–3695. DOI: 10.1002/pmic.200500924.

Wittenberg G, Järvi S, Hojka M, Tóth SZ, Meyer EH, Aro EM, Schöttler MA, Bock R (2017) Identification and characterization of a stable intermediate in photosystem I assembly in tobacco. *Plant J* 90: 478–490. DOI: 10.1111/tpj.13505
