# Supplementary figures and images for: STIC2 selectively binds ribosome-nascent chain complexes in the cotranslational sorting of Arabidopsis thylakoid proteins
Source: EMBO J. 2024 Aug 27;43(20):4699–719. doi: 10.1038/s44318-024-00211-4 (PMC11480477; doi:10.1038/s44318-024-00211-4)

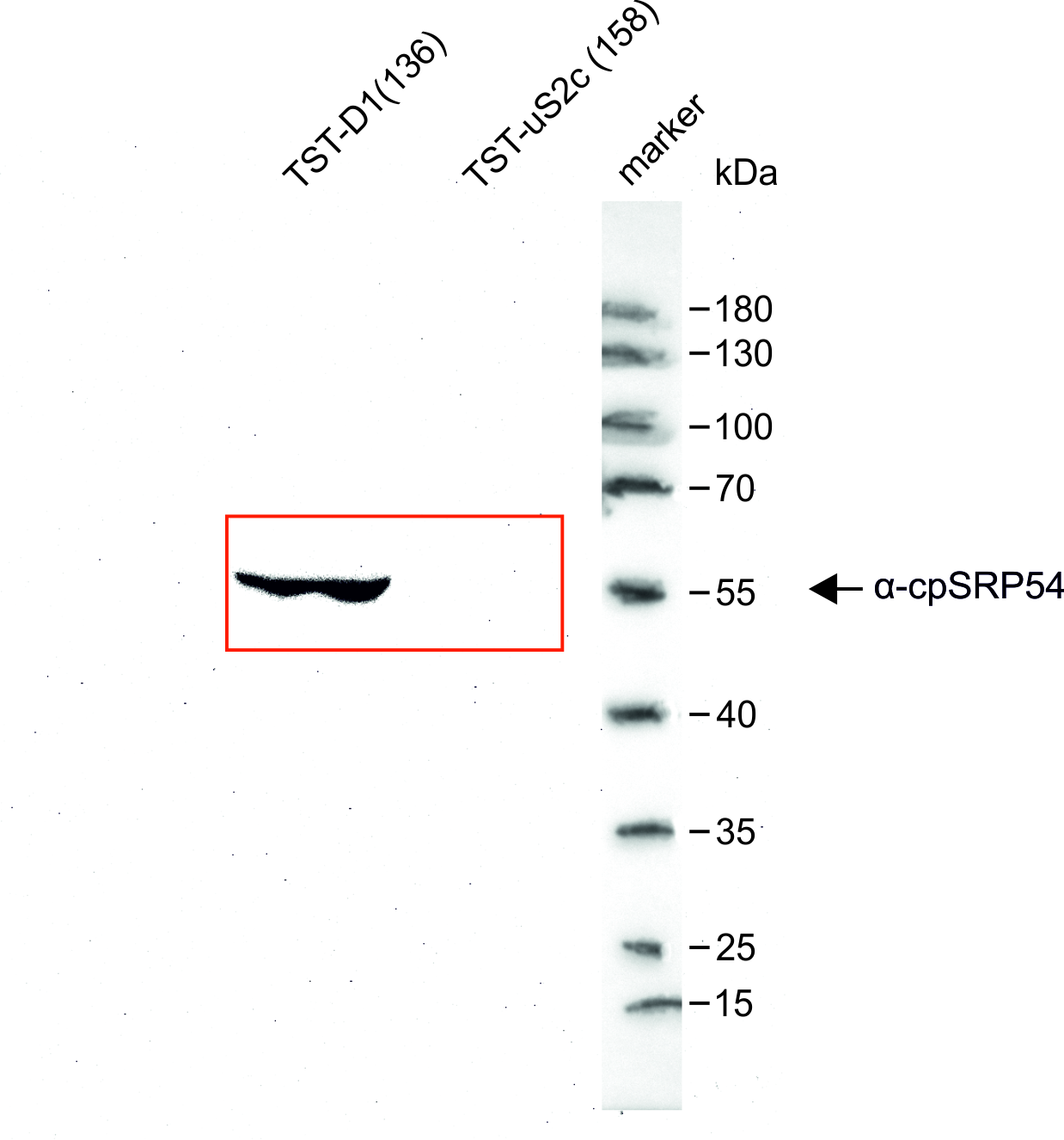

Supplement: Supplementary file 7 — Source data Fig. 2 [file 44318_2024_211_MOESM7_ESM.zip › Figure 2/2C/Western cpSRP54.tif]

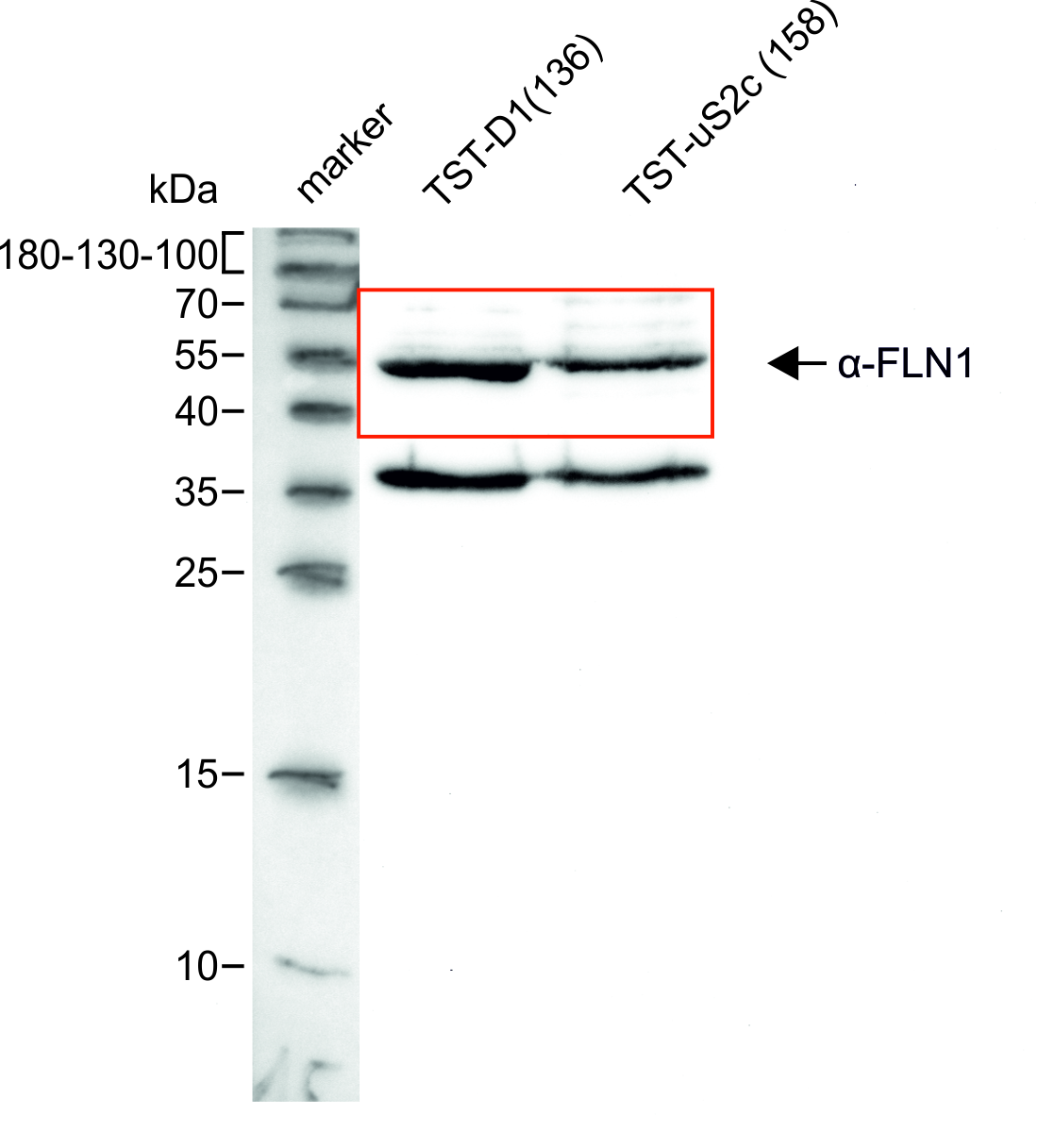

Supplement: Supplementary file 7 — Source data Fig. 2 [file 44318_2024_211_MOESM7_ESM.zip › Figure 2/2C/Western FLN1.tif]

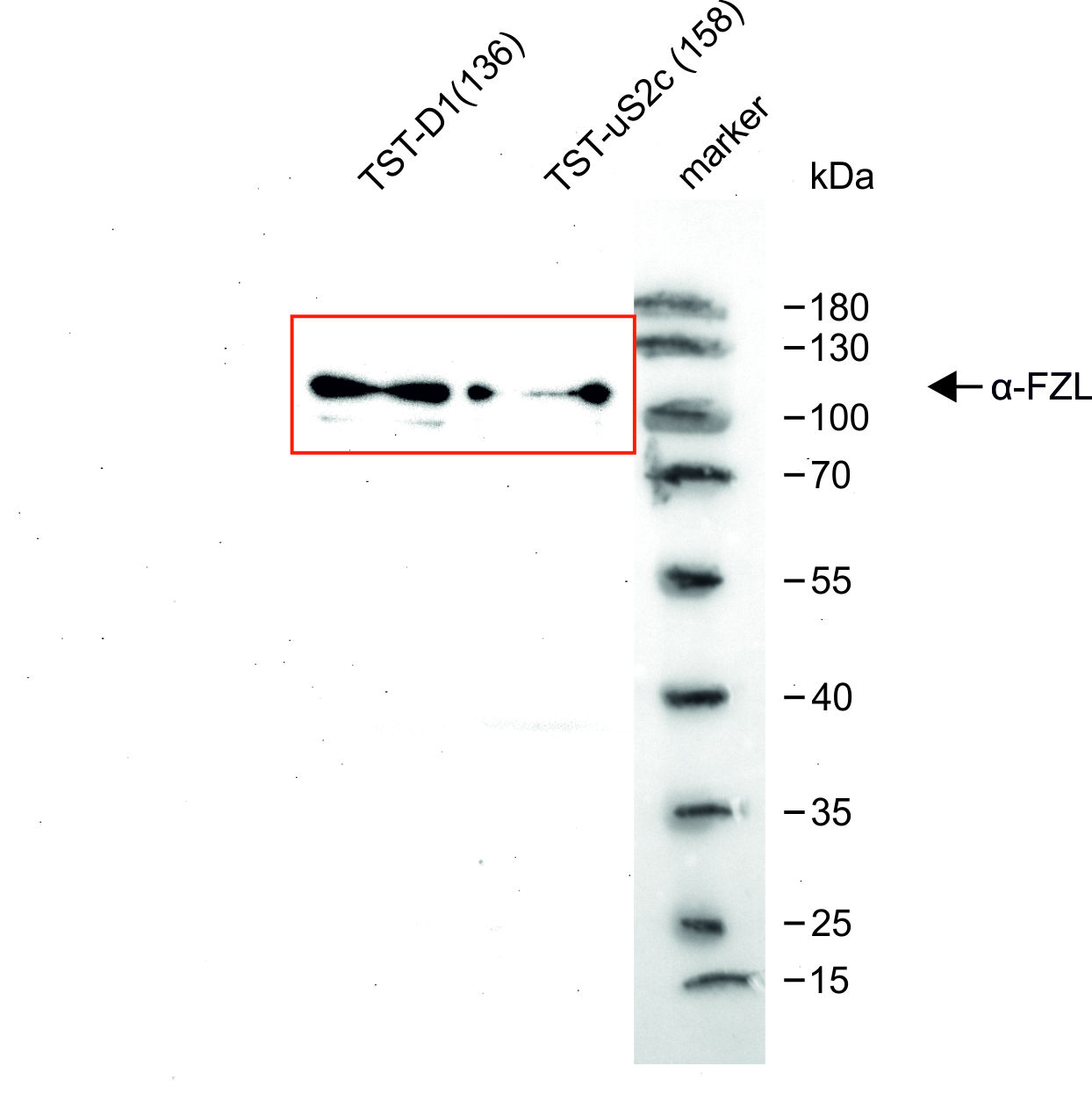

Supplement: Supplementary file 7 — Source data Fig. 2 [file 44318_2024_211_MOESM7_ESM.zip › Figure 2/2C/Western FZL.tif]

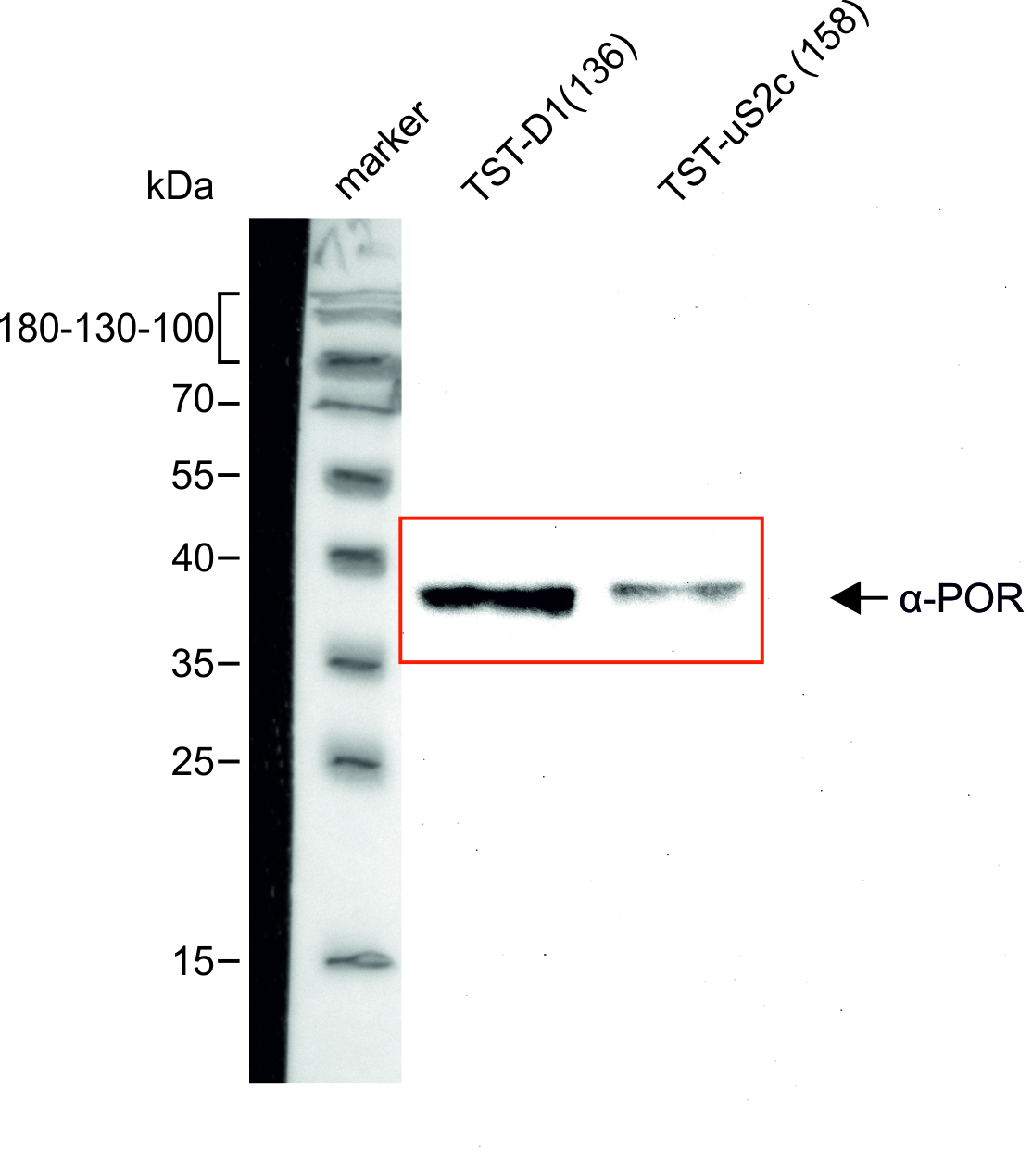

Supplement: Supplementary file 7 — Source data Fig. 2 [file 44318_2024_211_MOESM7_ESM.zip › Figure 2/2C/Western POR.tif]

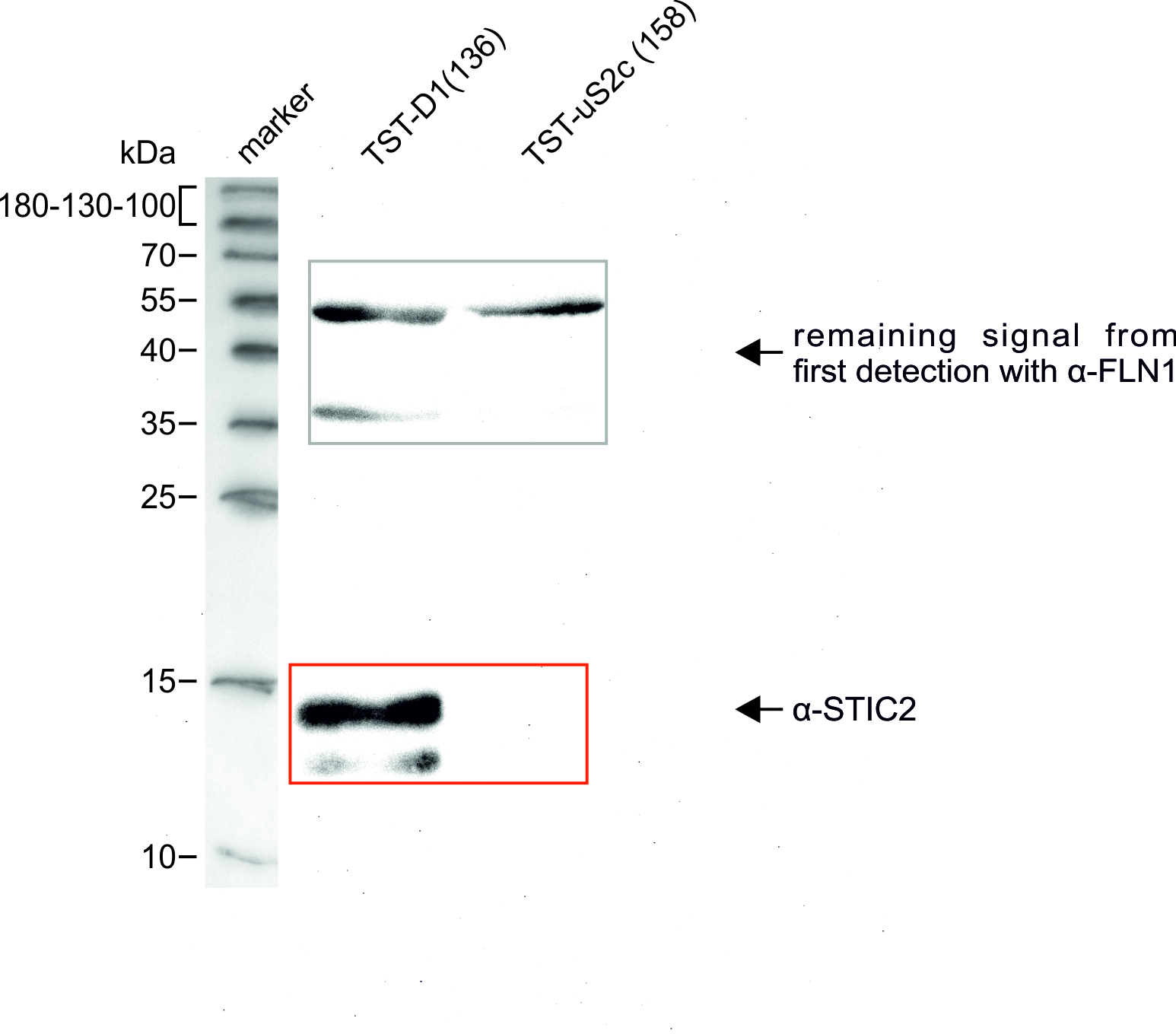

Supplement: Supplementary file 7 — Source data Fig. 2 [file 44318_2024_211_MOESM7_ESM.zip › Figure 2/2C/Western STIC2.tif]

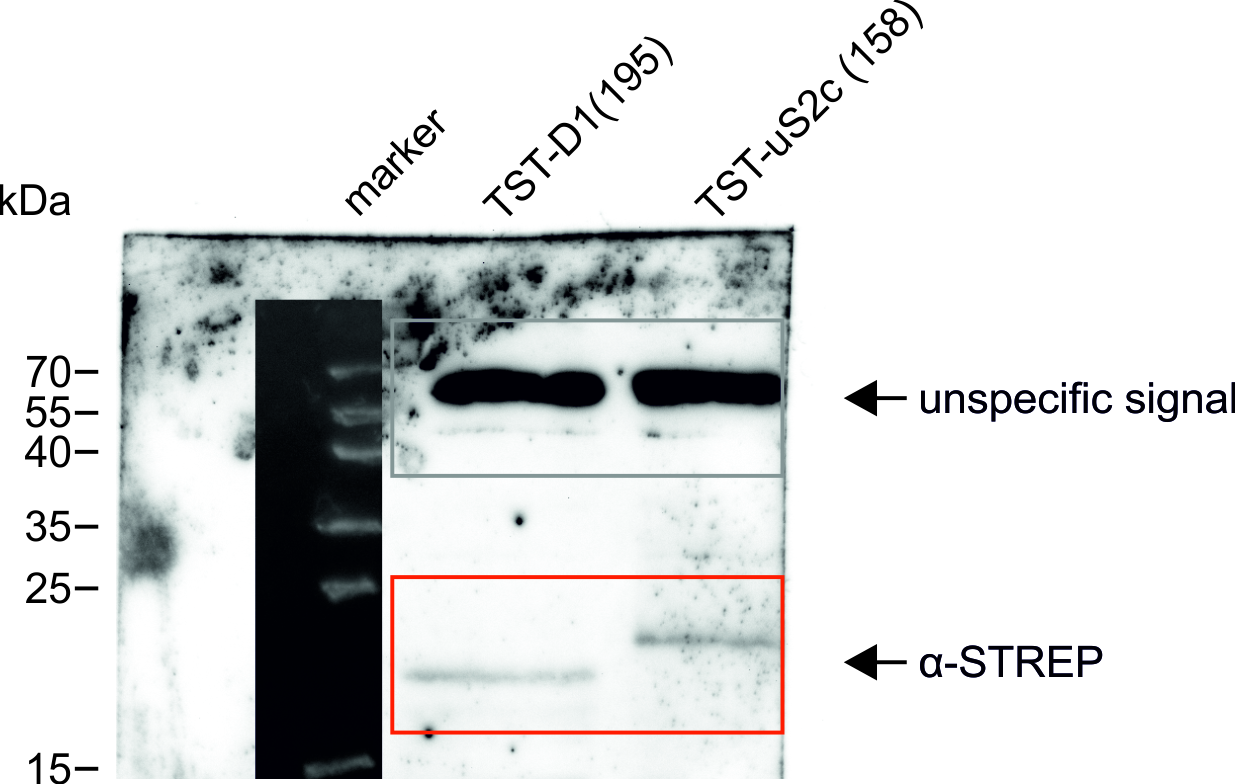

Supplement: Supplementary file 7 — Source data Fig. 2 [file 44318_2024_211_MOESM7_ESM.zip › Figure 2/2C/Western STREP.tif]

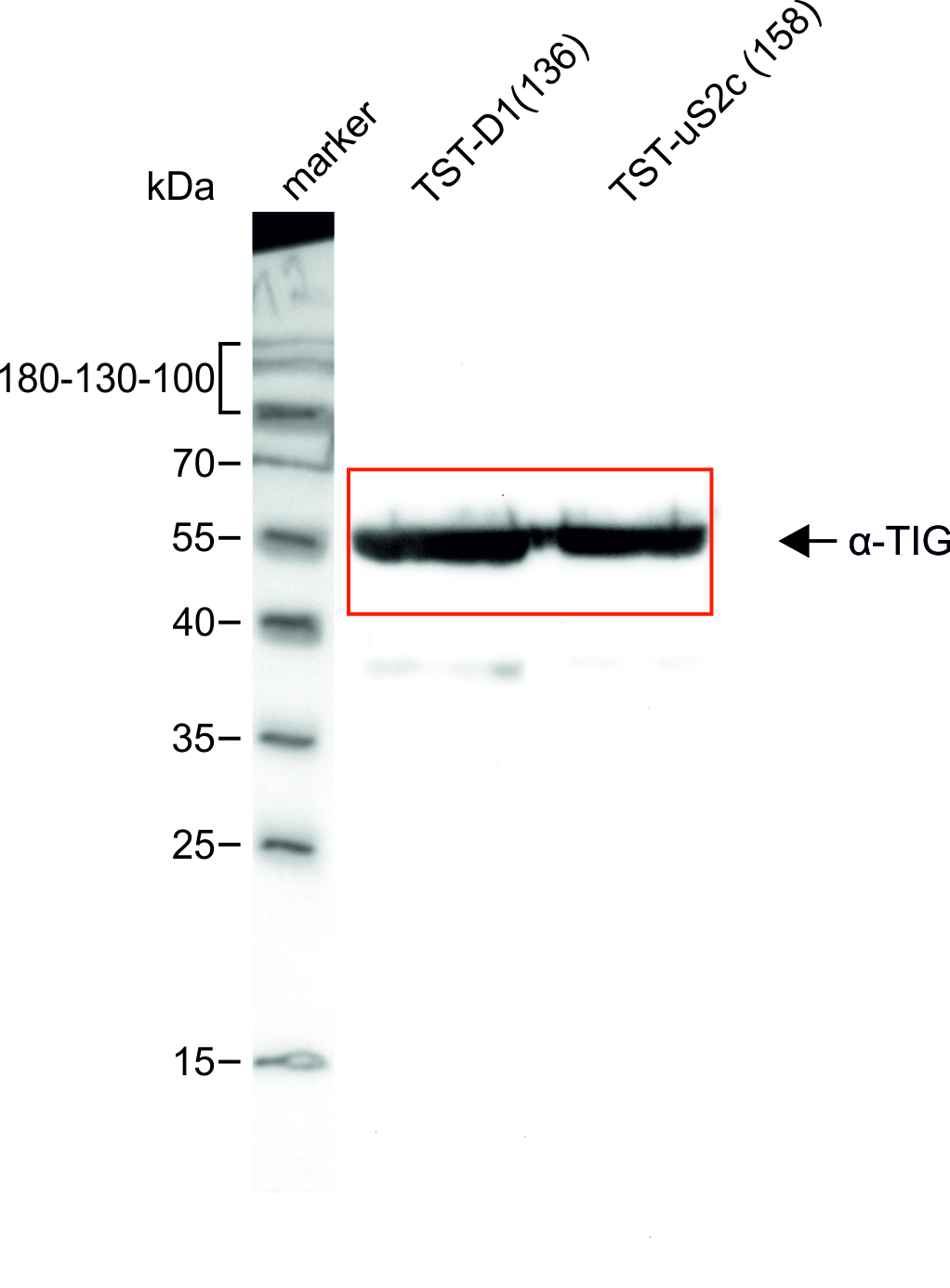

Supplement: Supplementary file 7 — Source data Fig. 2 [file 44318_2024_211_MOESM7_ESM.zip › Figure 2/2C/Western TIG.tif]

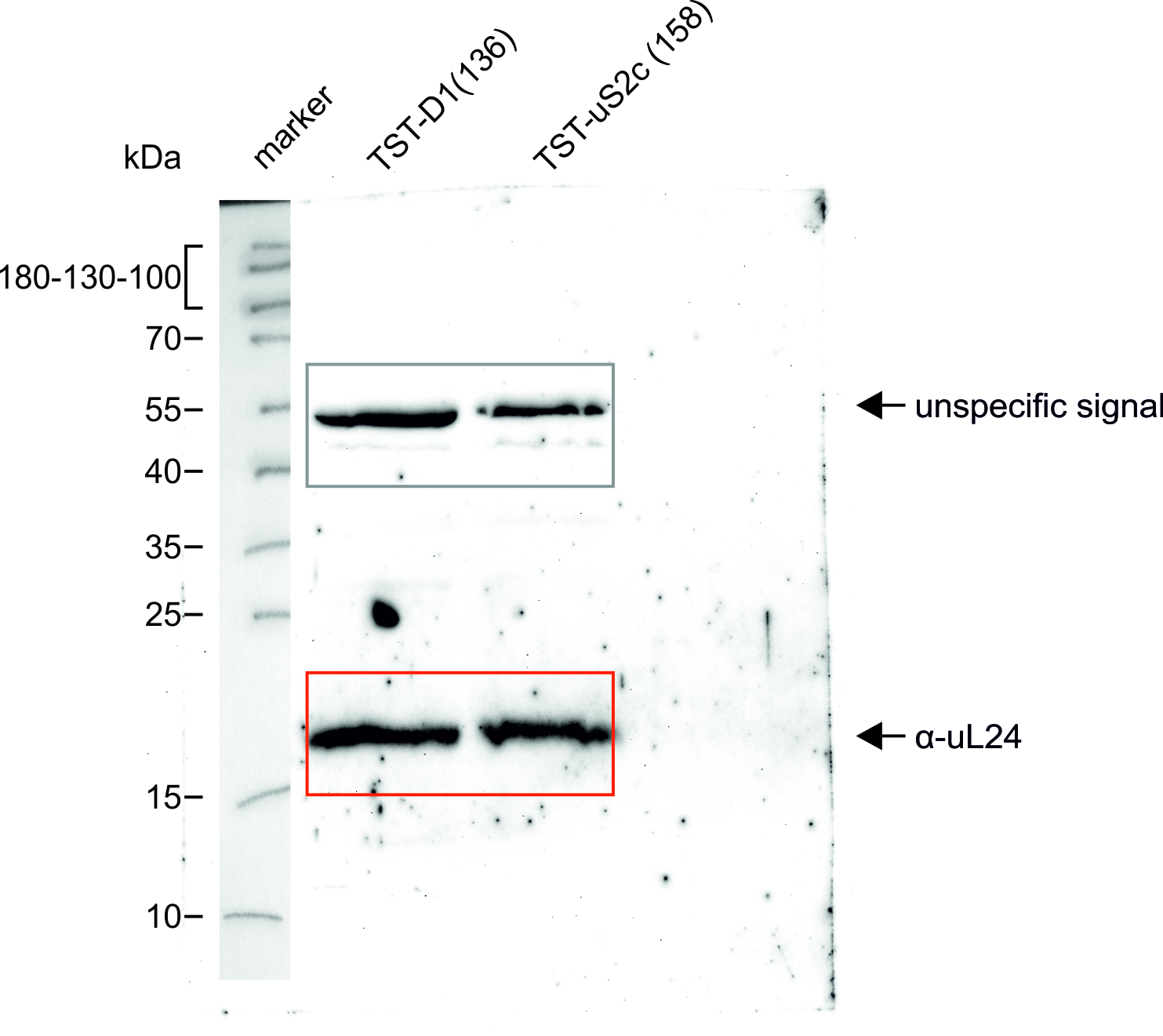

Supplement: Supplementary file 7 — Source data Fig. 2 [file 44318_2024_211_MOESM7_ESM.zip › Figure 2/2C/Western uL24.tif]

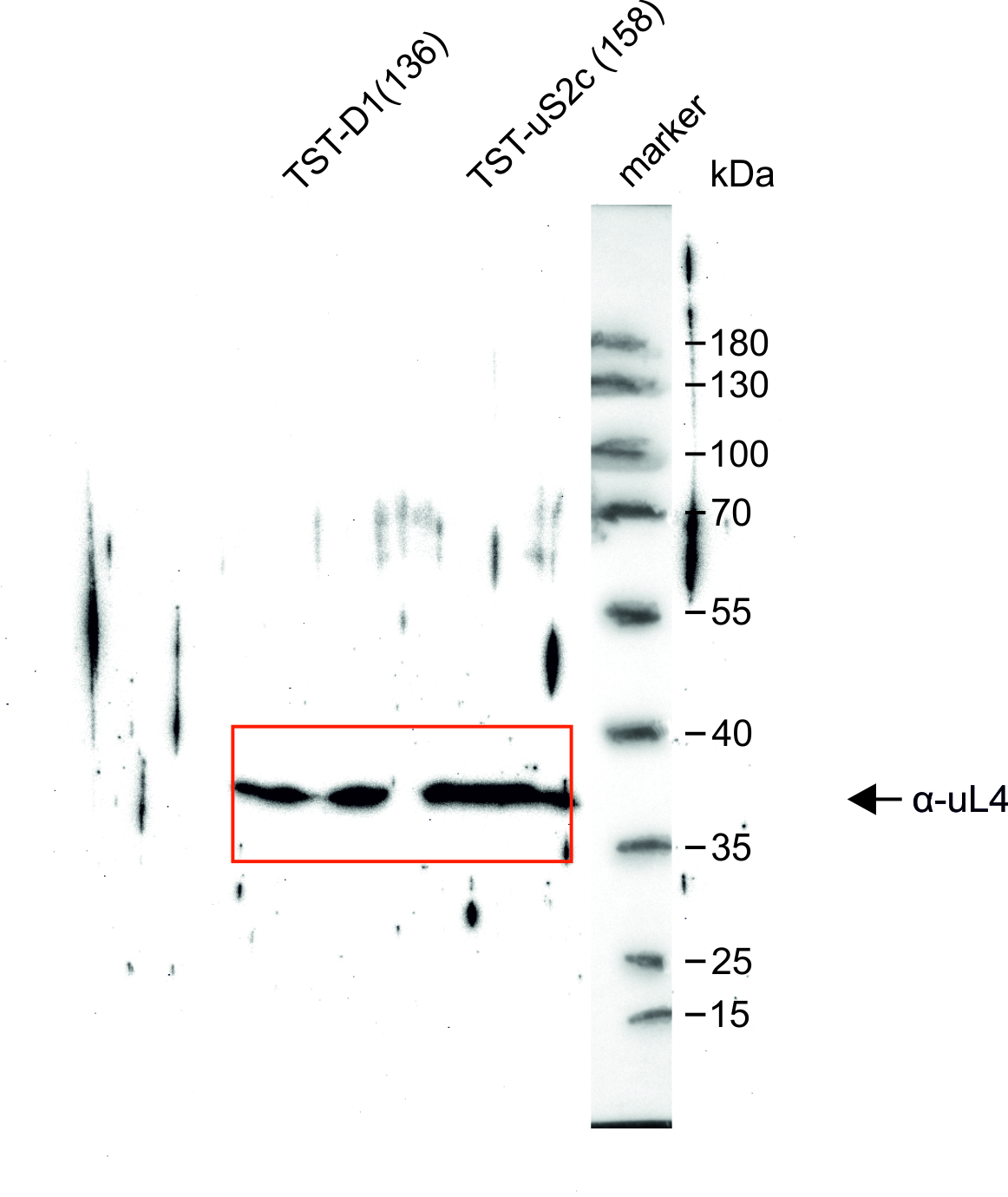

Supplement: Supplementary file 7 — Source data Fig. 2 [file 44318_2024_211_MOESM7_ESM.zip › Figure 2/2C/Western uL4.tif]

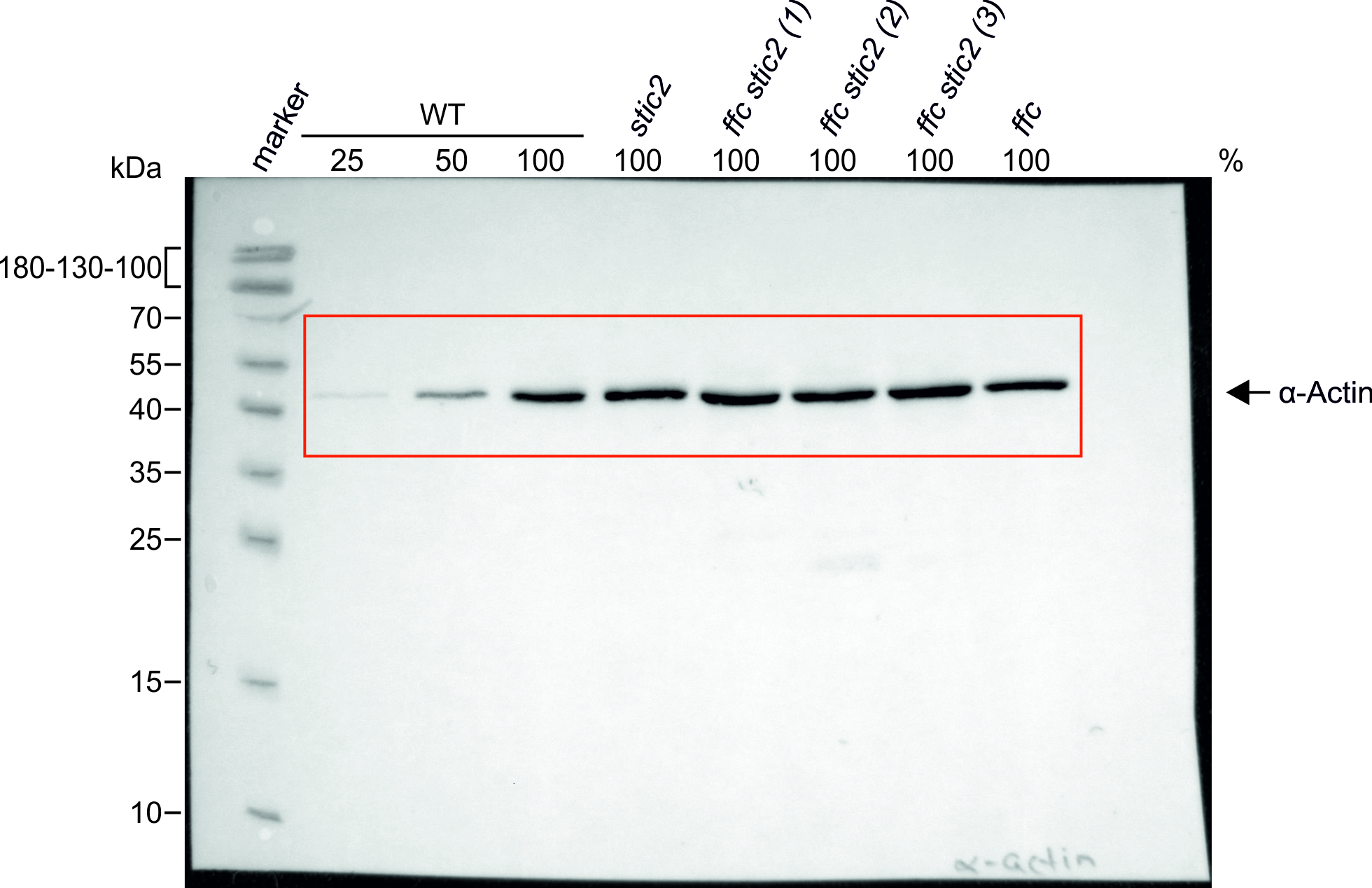

Supplement: Supplementary file 8 — Source data Fig. 3 [file 44318_2024_211_MOESM8_ESM.zip › Figure 3/3B/Western Actin.tif]

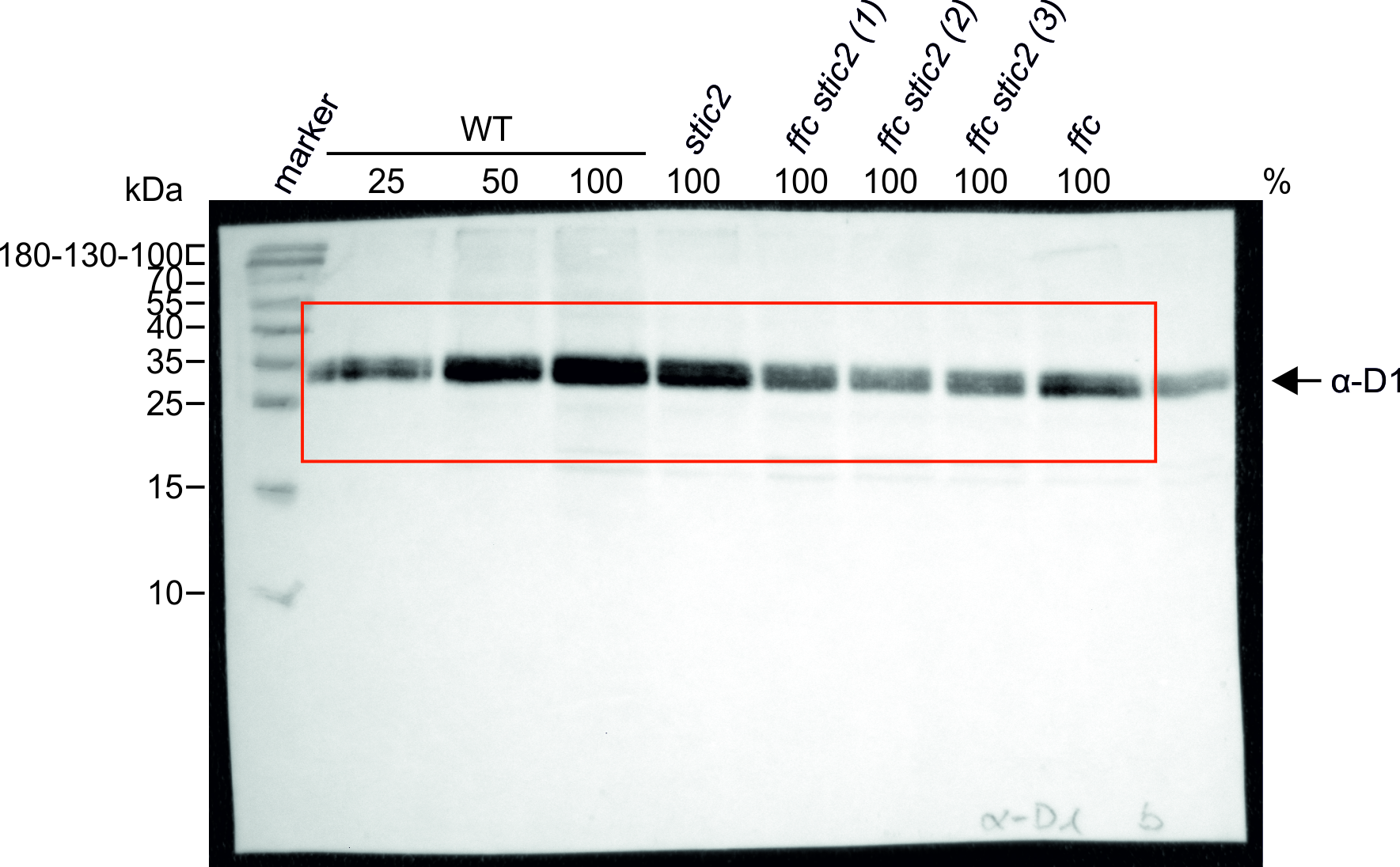

Supplement: Supplementary file 8 — Source data Fig. 3 [file 44318_2024_211_MOESM8_ESM.zip › Figure 3/3B/Western D1.tif]

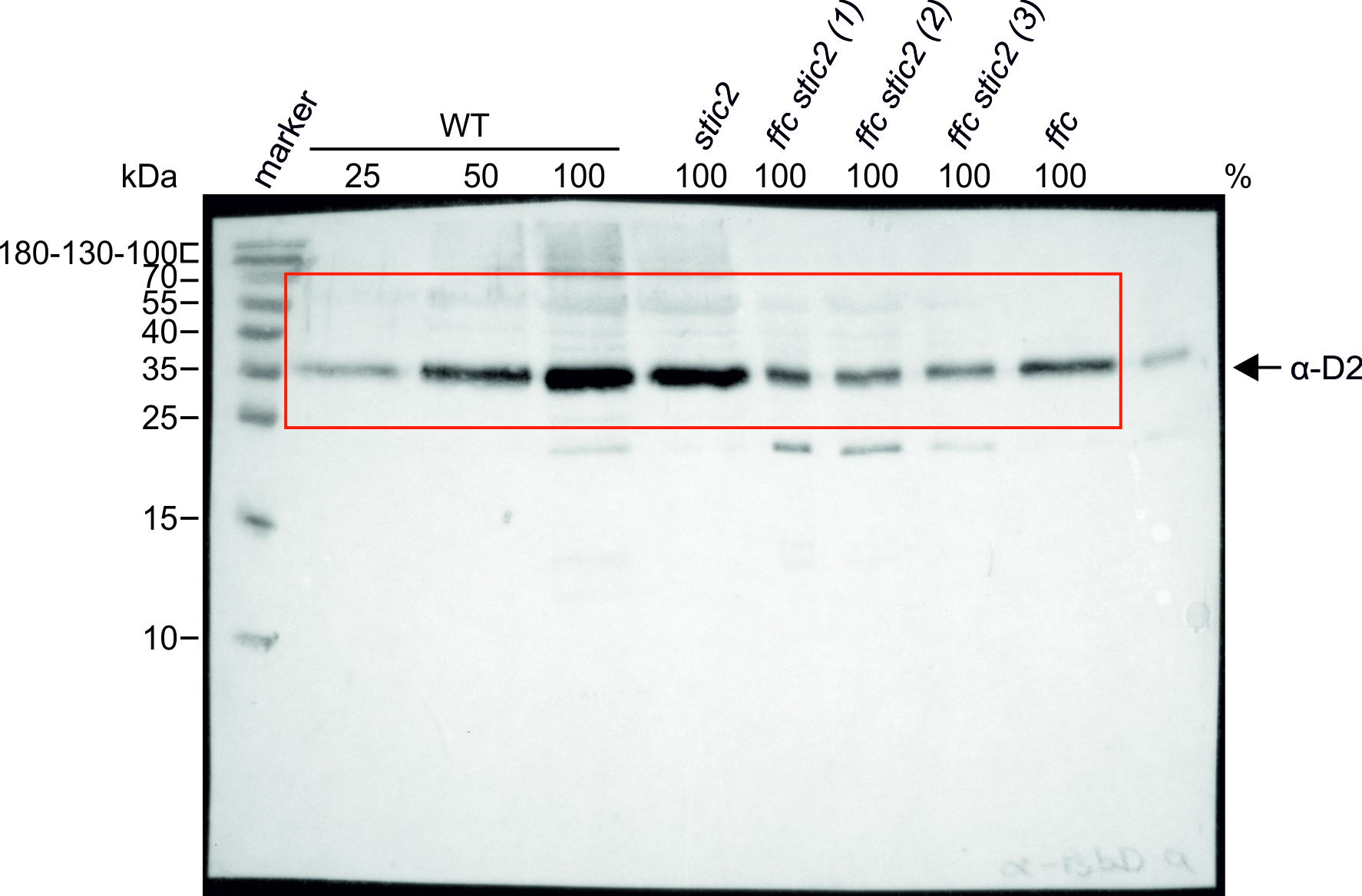

Supplement: Supplementary file 8 — Source data Fig. 3 [file 44318_2024_211_MOESM8_ESM.zip › Figure 3/3B/Western D2.tif]

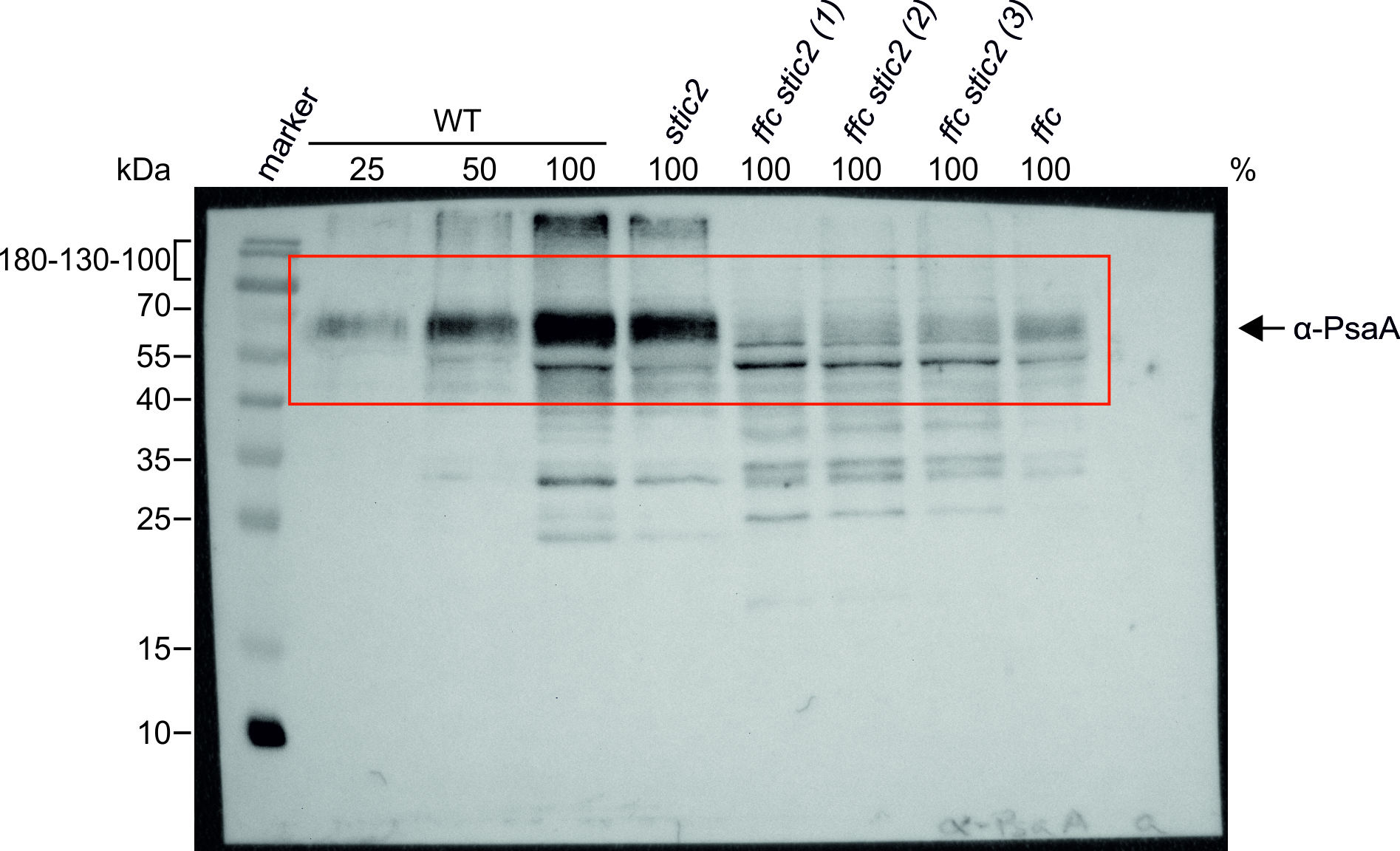

Supplement: Supplementary file 8 — Source data Fig. 3 [file 44318_2024_211_MOESM8_ESM.zip › Figure 3/3B/Western PsaA.tif]

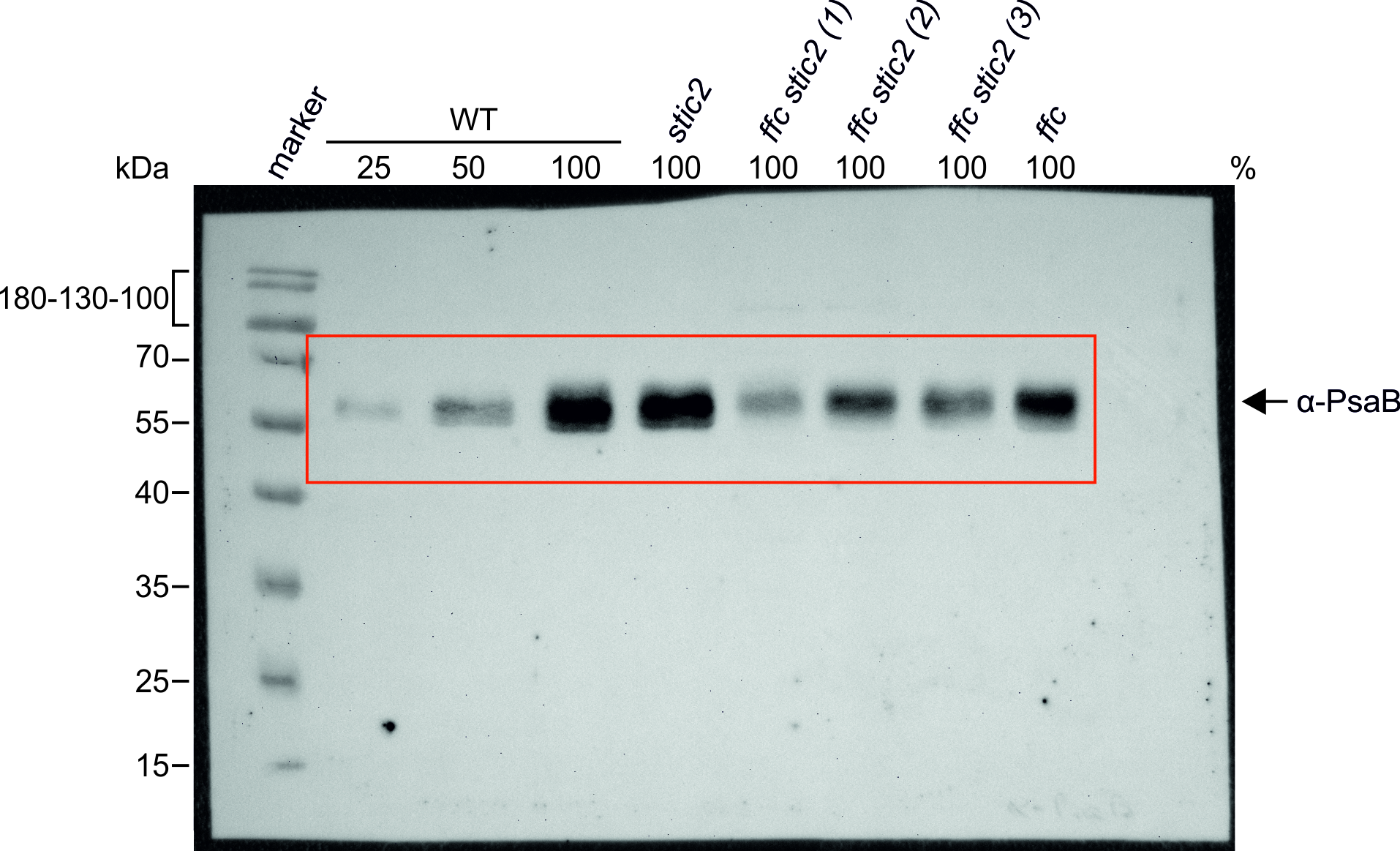

Supplement: Supplementary file 8 — Source data Fig. 3 [file 44318_2024_211_MOESM8_ESM.zip › Figure 3/3B/Western PsaB.tif]

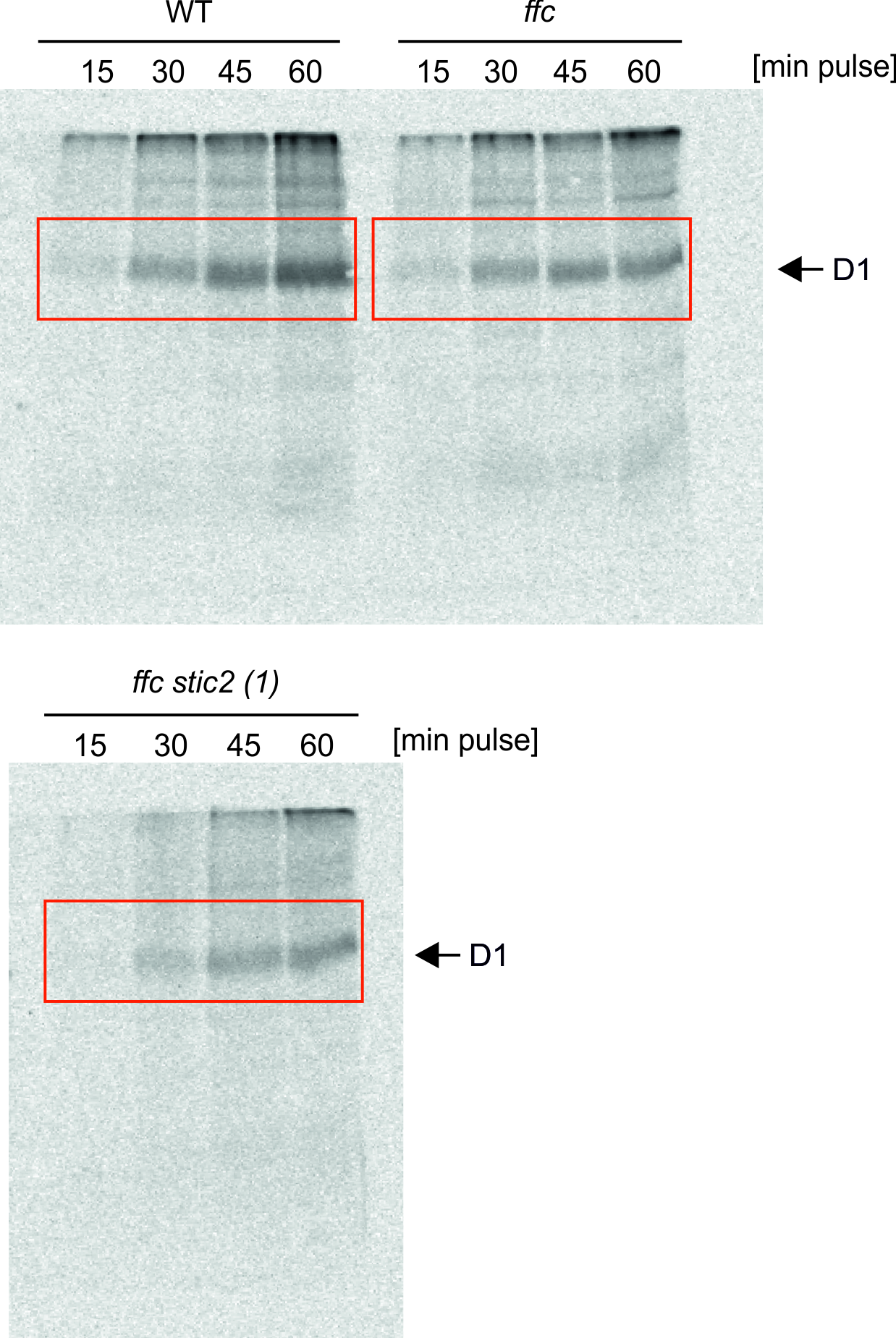

Supplement: Supplementary file 8 — Source data Fig. 3 [file 44318_2024_211_MOESM8_ESM.zip › Figure 3/3C/Phospho Image left panel.tif]

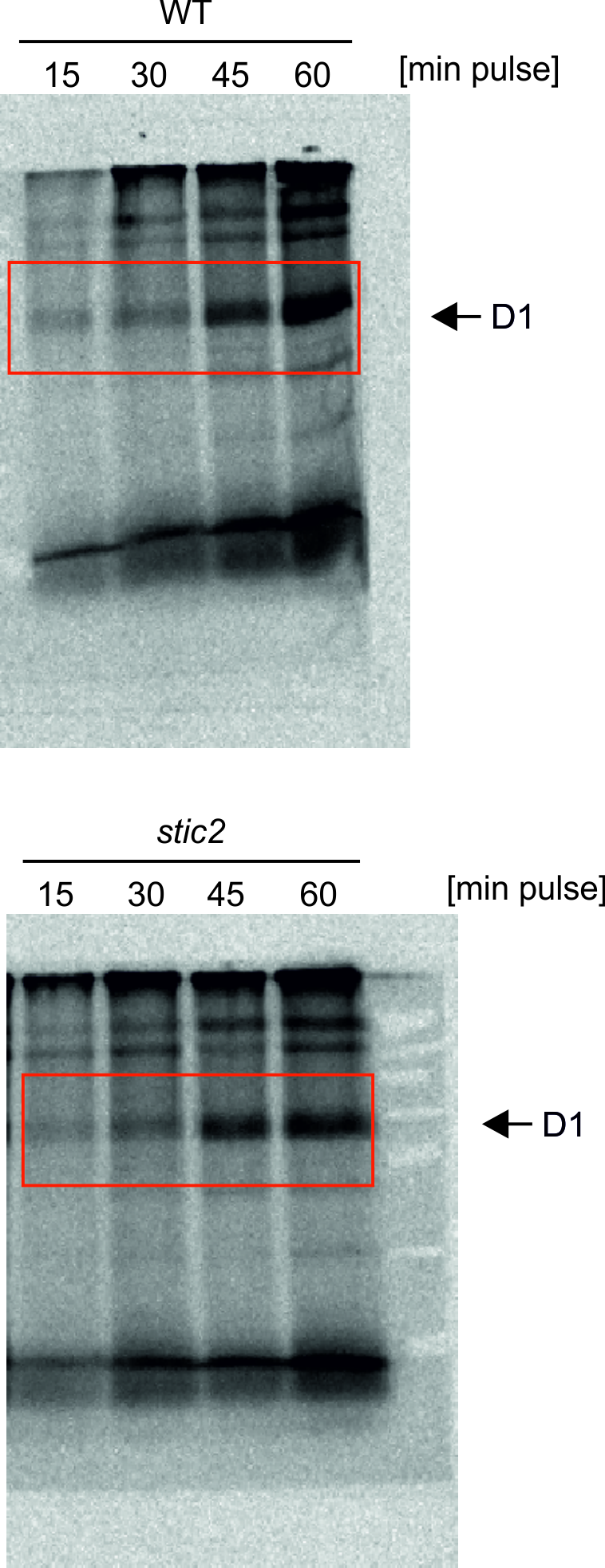

Supplement: Supplementary file 8 — Source data Fig. 3 [file 44318_2024_211_MOESM8_ESM.zip › Figure 3/3C/Phospho Image right panel.tif]

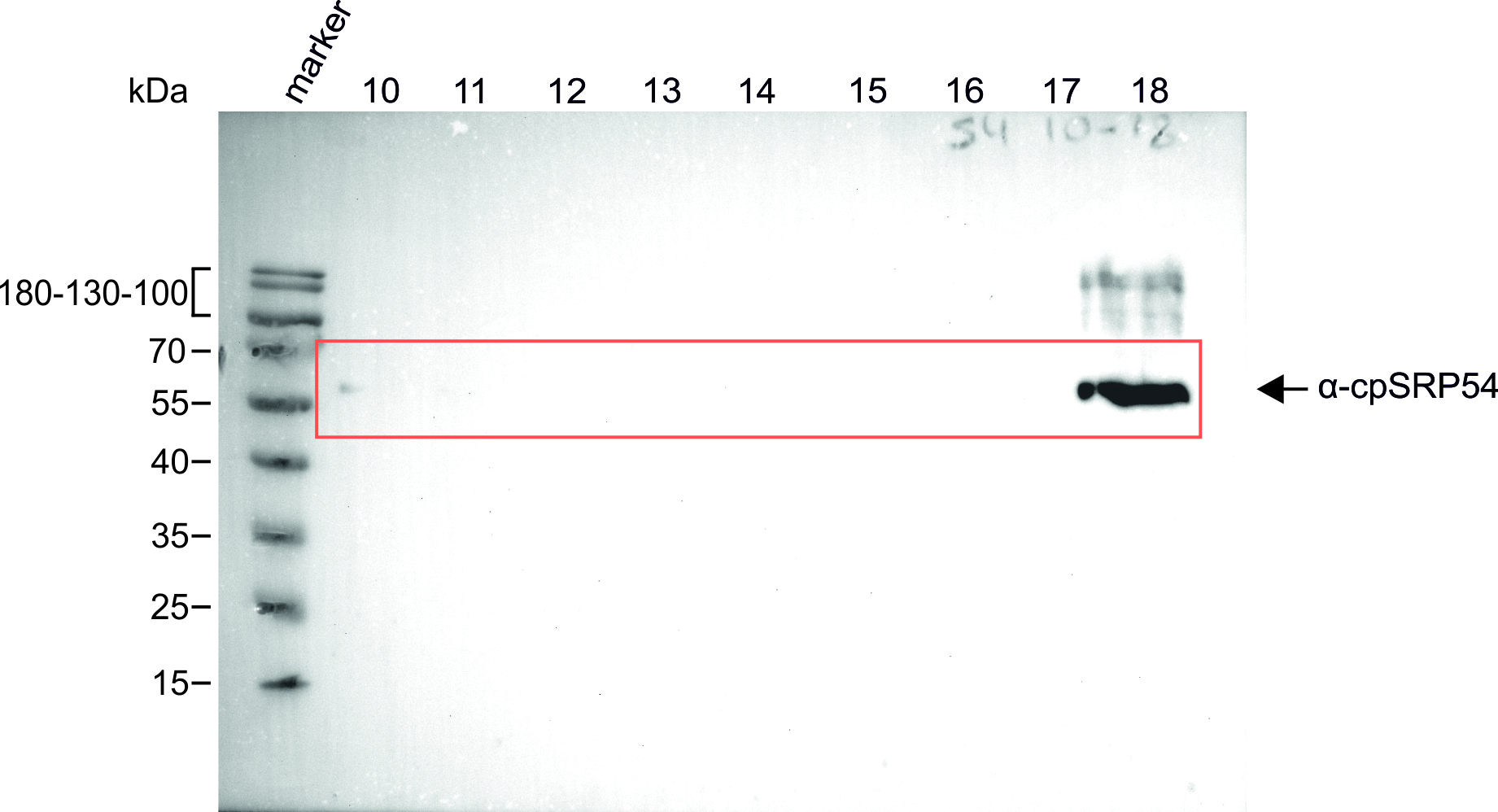

Supplement: Supplementary file 9 — Source data Fig. 4 [file 44318_2024_211_MOESM9_ESM.zip › Figure 4/4A/Western cpSRP54 fractions 10-18.tif]

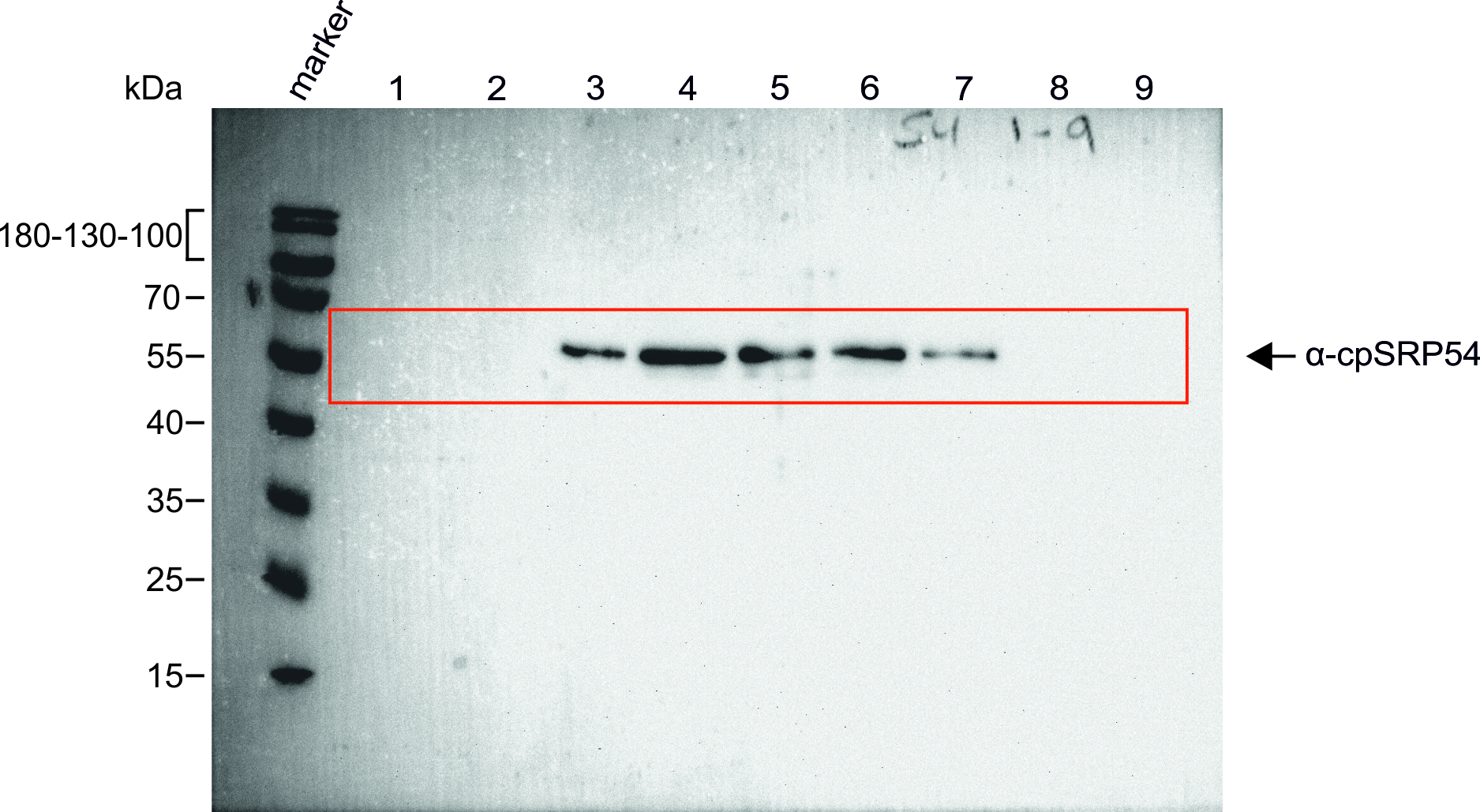

Supplement: Supplementary file 9 — Source data Fig. 4 [file 44318_2024_211_MOESM9_ESM.zip › Figure 4/4A/Western cpSRP54 fractions 1-9.tif]

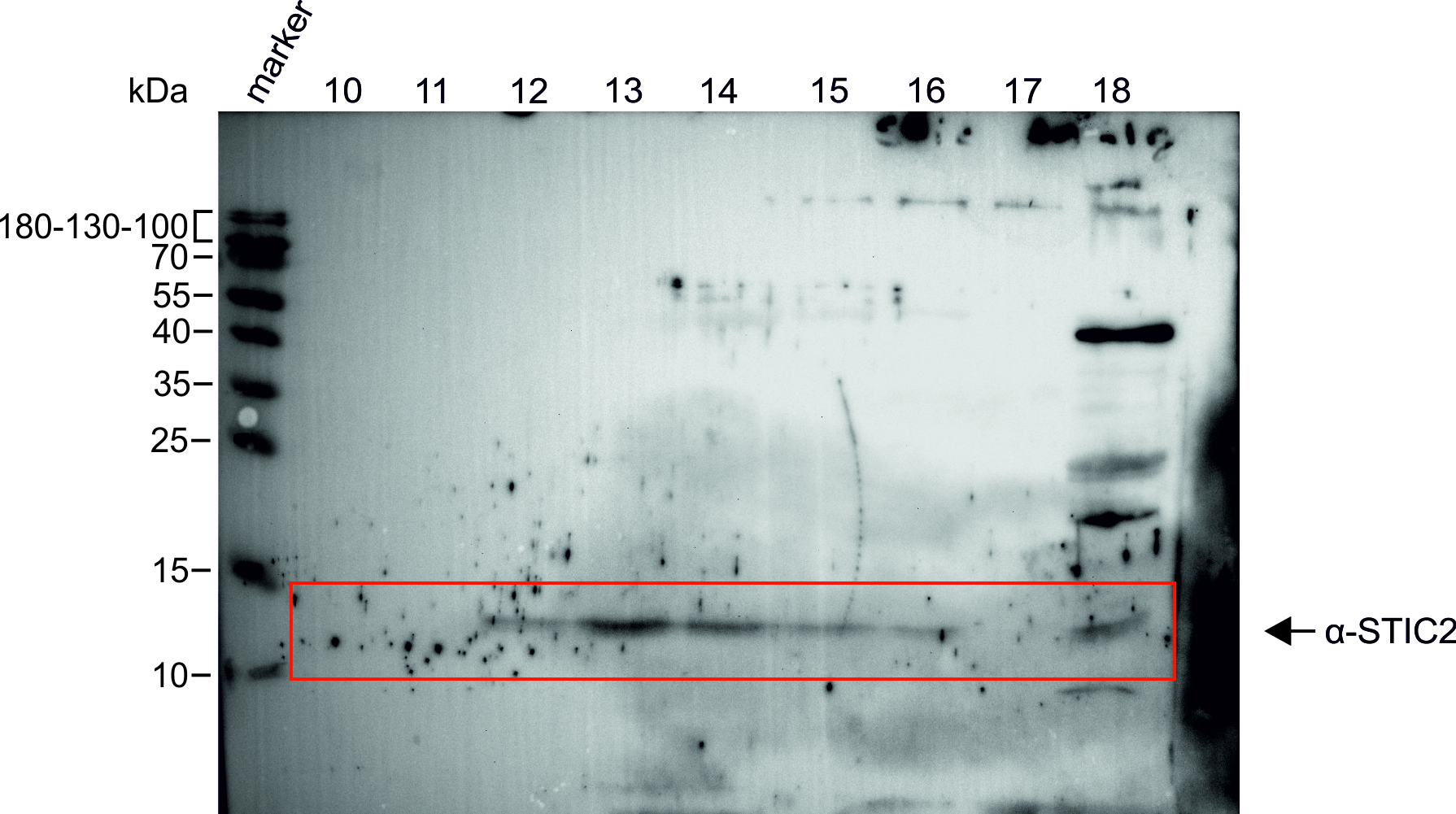

Supplement: Supplementary file 9 — Source data Fig. 4 [file 44318_2024_211_MOESM9_ESM.zip › Figure 4/4A/Western STIC2 fractions 10-18.tif]

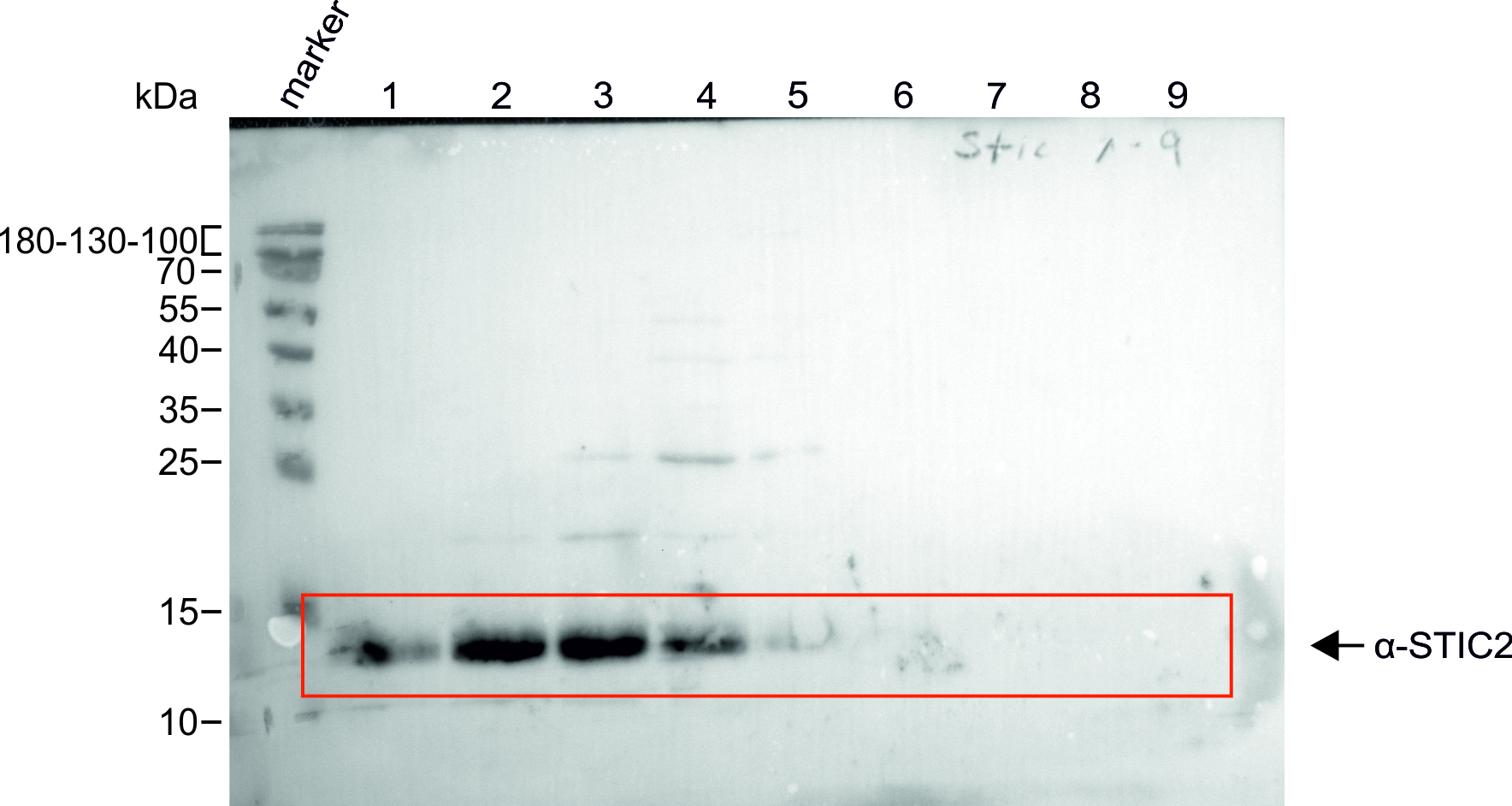

Supplement: Supplementary file 9 — Source data Fig. 4 [file 44318_2024_211_MOESM9_ESM.zip › Figure 4/4A/Western STIC2 fractions 1-9.tif]

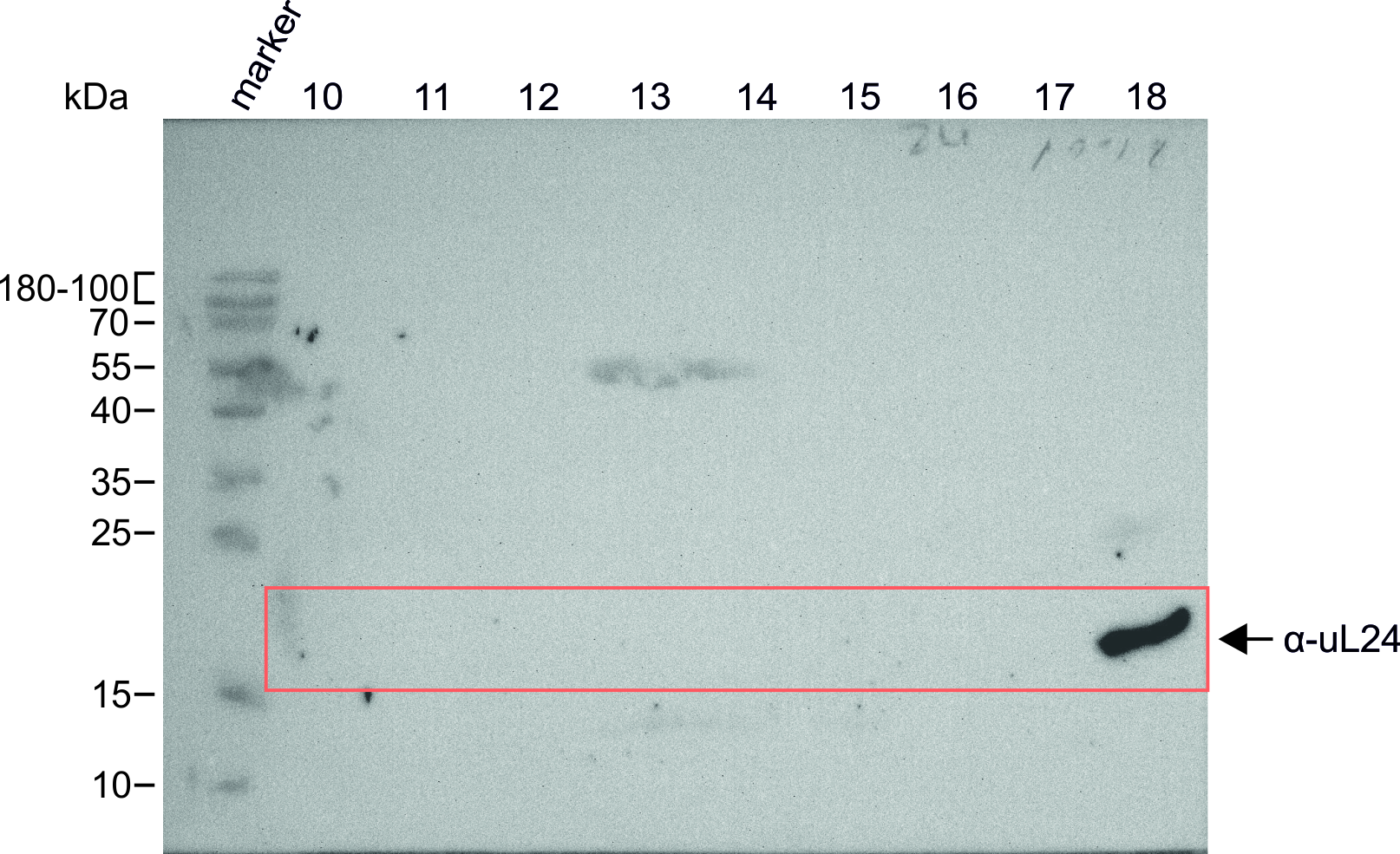

Supplement: Supplementary file 9 — Source data Fig. 4 [file 44318_2024_211_MOESM9_ESM.zip › Figure 4/4A/Western uL24 fractions 10-18.tif]

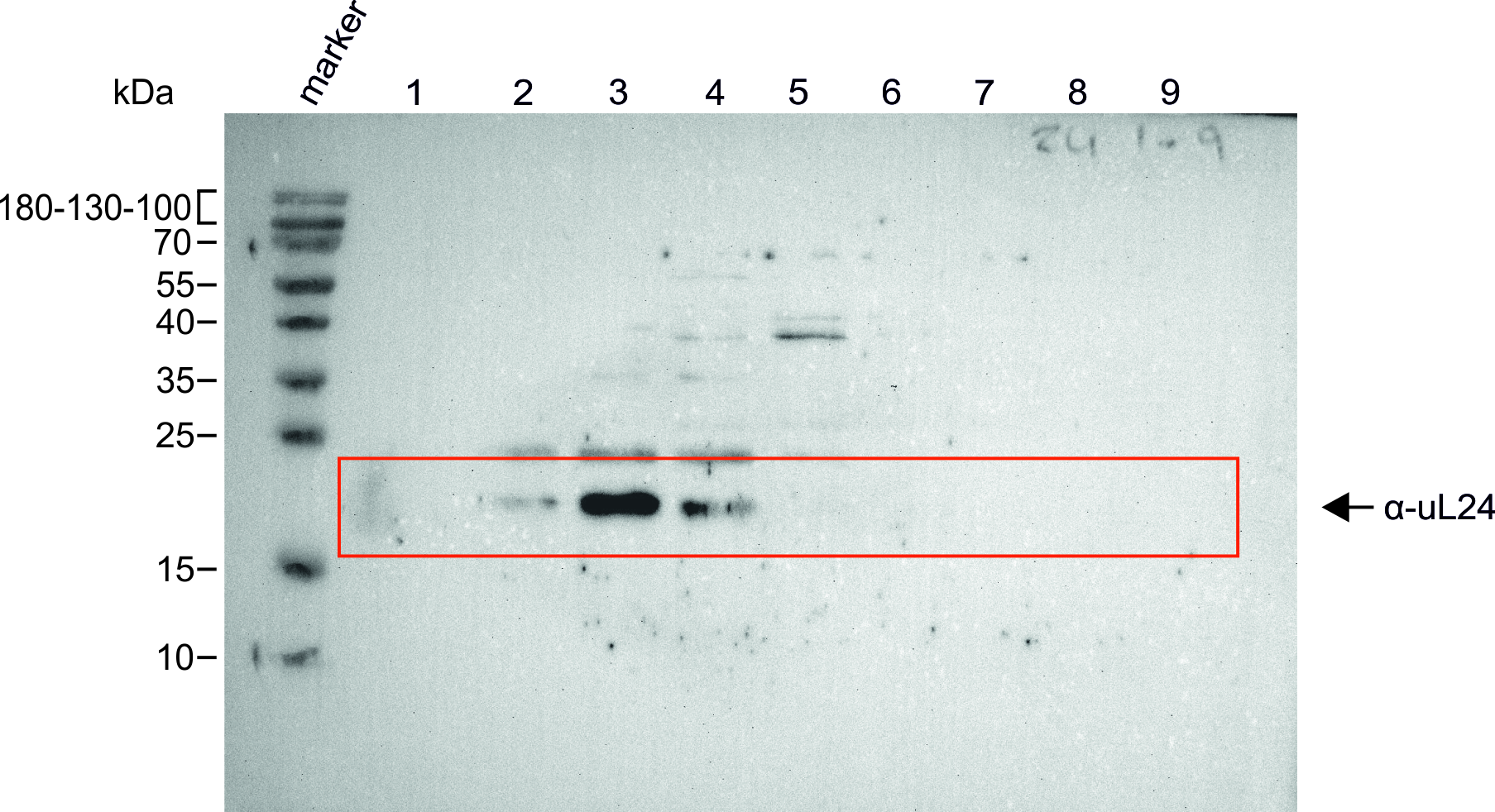

Supplement: Supplementary file 9 — Source data Fig. 4 [file 44318_2024_211_MOESM9_ESM.zip › Figure 4/4A/Western uL24 fractions 1-9.tif]

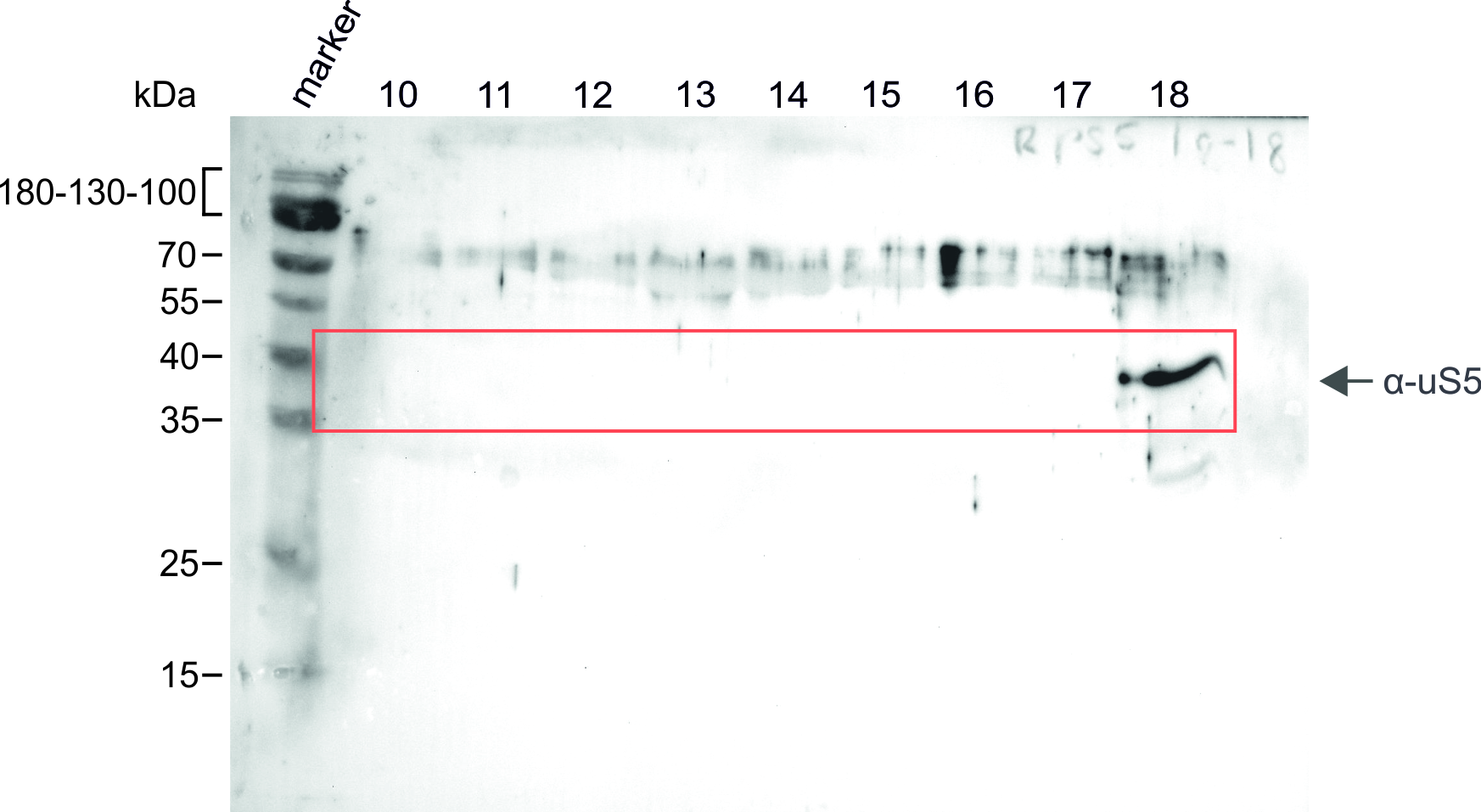

Supplement: Supplementary file 9 — Source data Fig. 4 [file 44318_2024_211_MOESM9_ESM.zip › Figure 4/4A/Western uS5 fractions 10-18.tif]

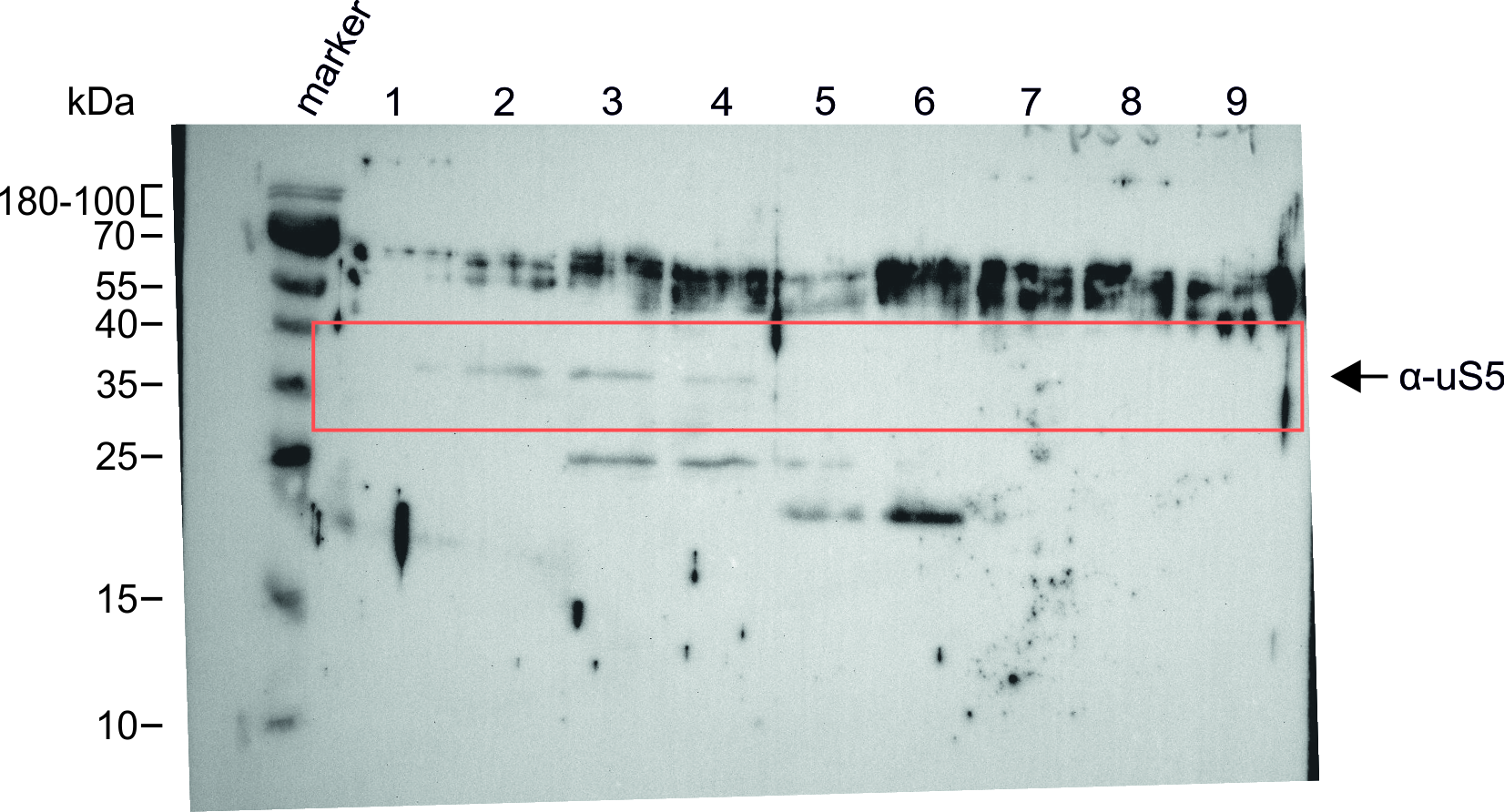

Supplement: Supplementary file 9 — Source data Fig. 4 [file 44318_2024_211_MOESM9_ESM.zip › Figure 4/4A/Western uS5 fractions 1-9.tif]

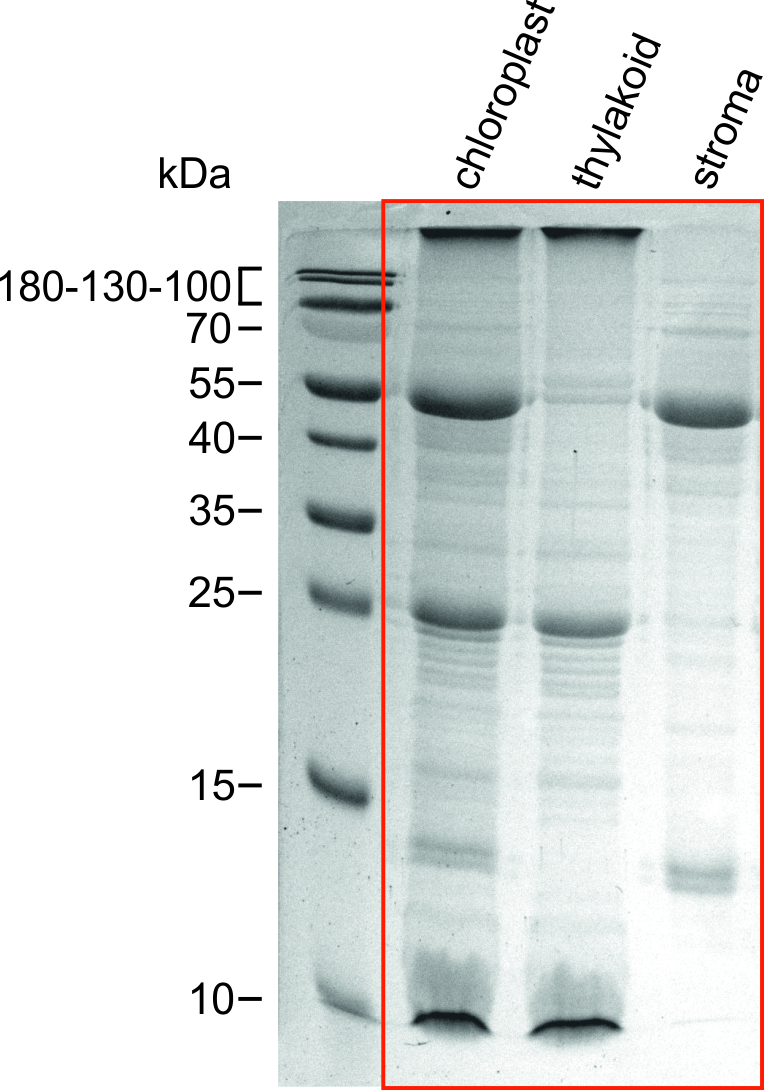

Supplement: Supplementary file 9 — Source data Fig. 4 [file 44318_2024_211_MOESM9_ESM.zip › Figure 4/4B/Coomassie.tif]

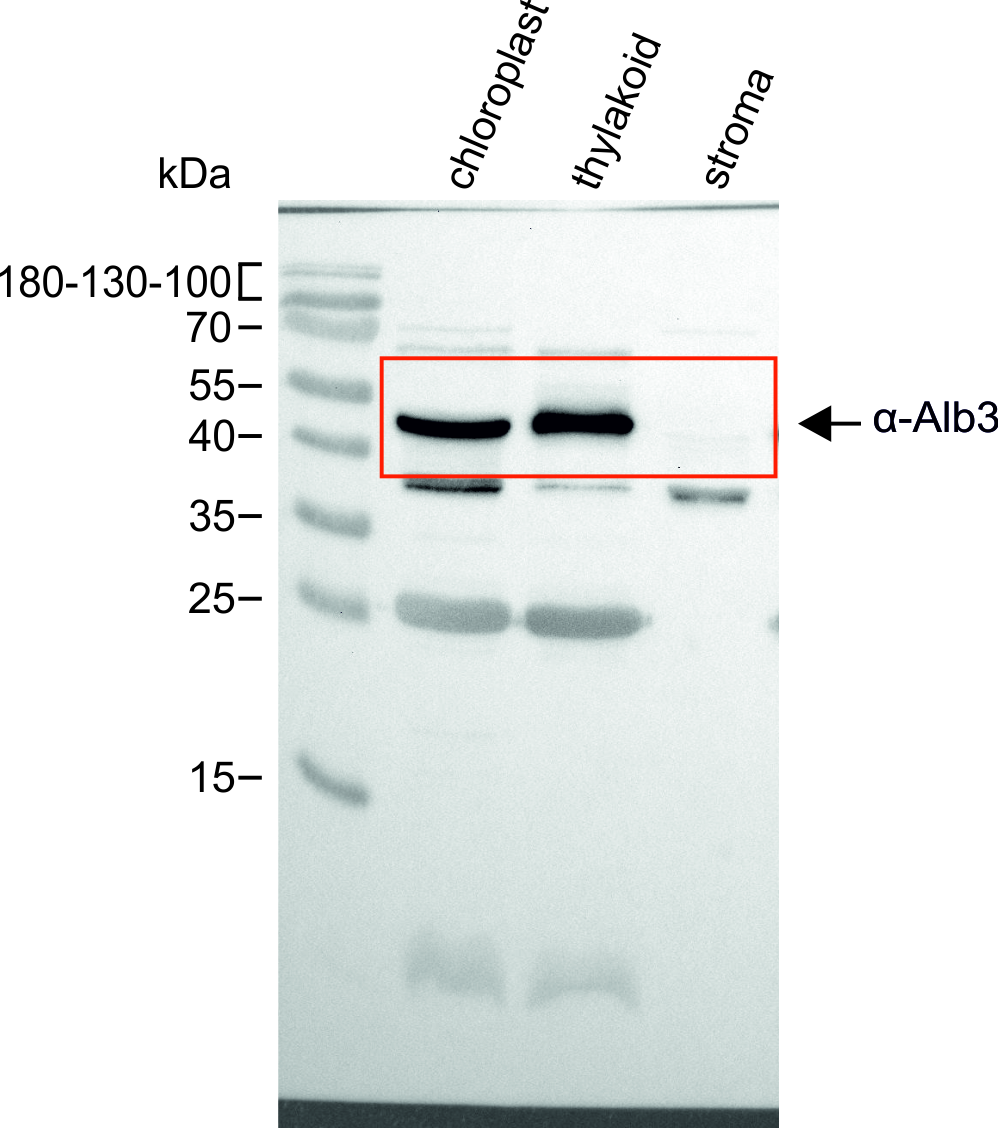

Supplement: Supplementary file 9 — Source data Fig. 4 [file 44318_2024_211_MOESM9_ESM.zip › Figure 4/4B/Western Alb3.tif]

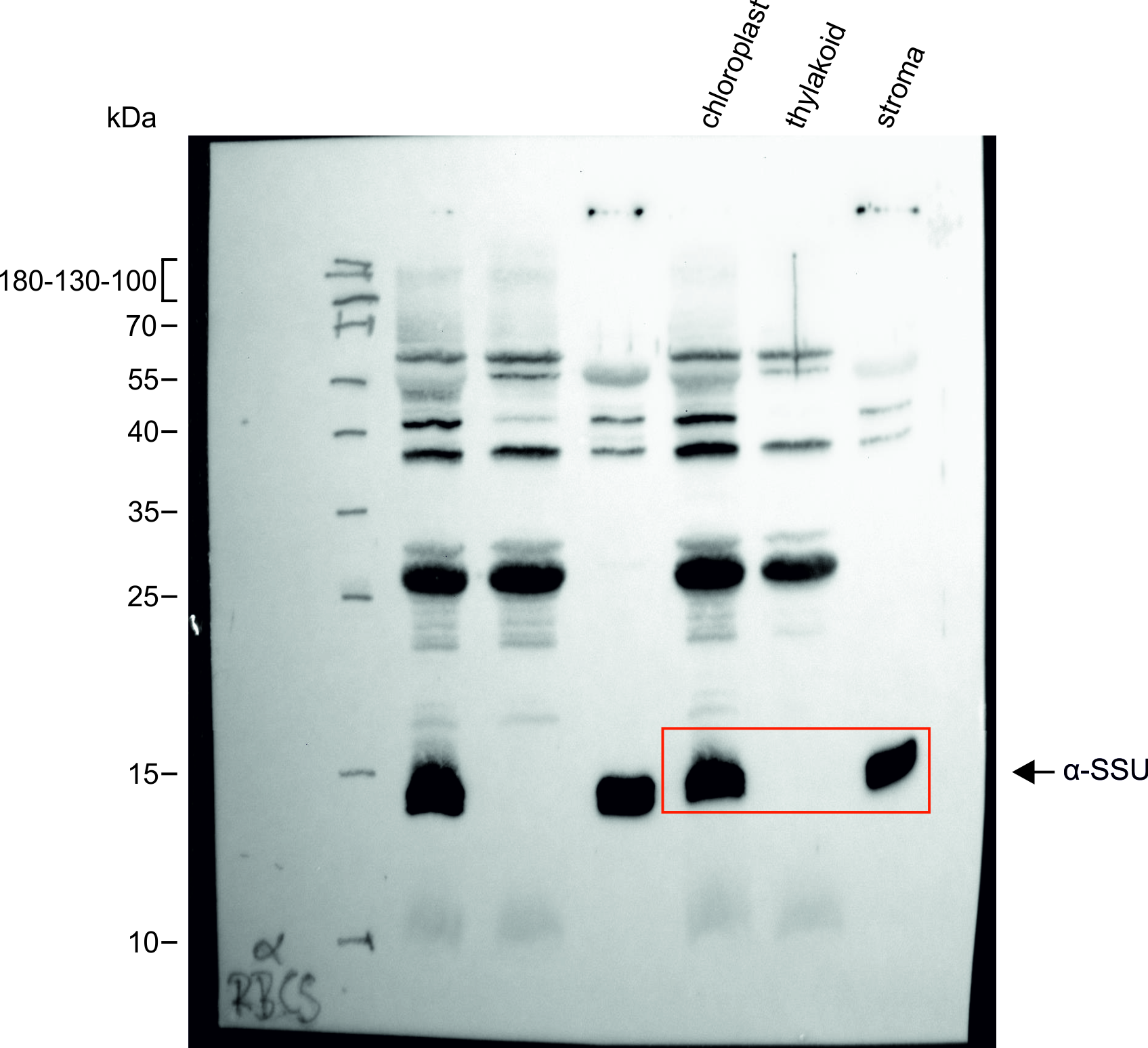

Supplement: Supplementary file 9 — Source data Fig. 4 [file 44318_2024_211_MOESM9_ESM.zip › Figure 4/4B/Western SSU.tif]

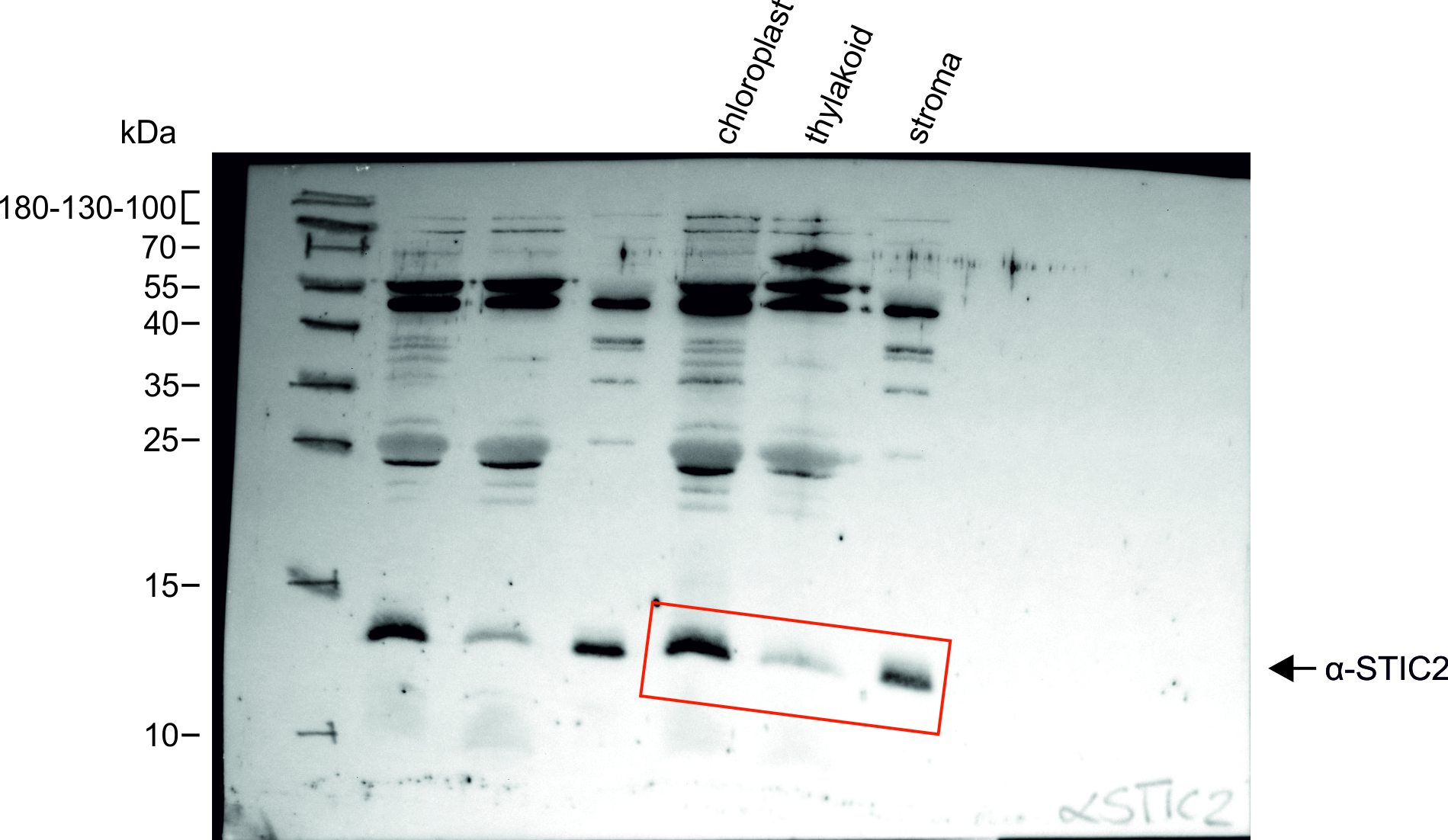

Supplement: Supplementary file 9 — Source data Fig. 4 [file 44318_2024_211_MOESM9_ESM.zip › Figure 4/4B/Western STIC2.tif]

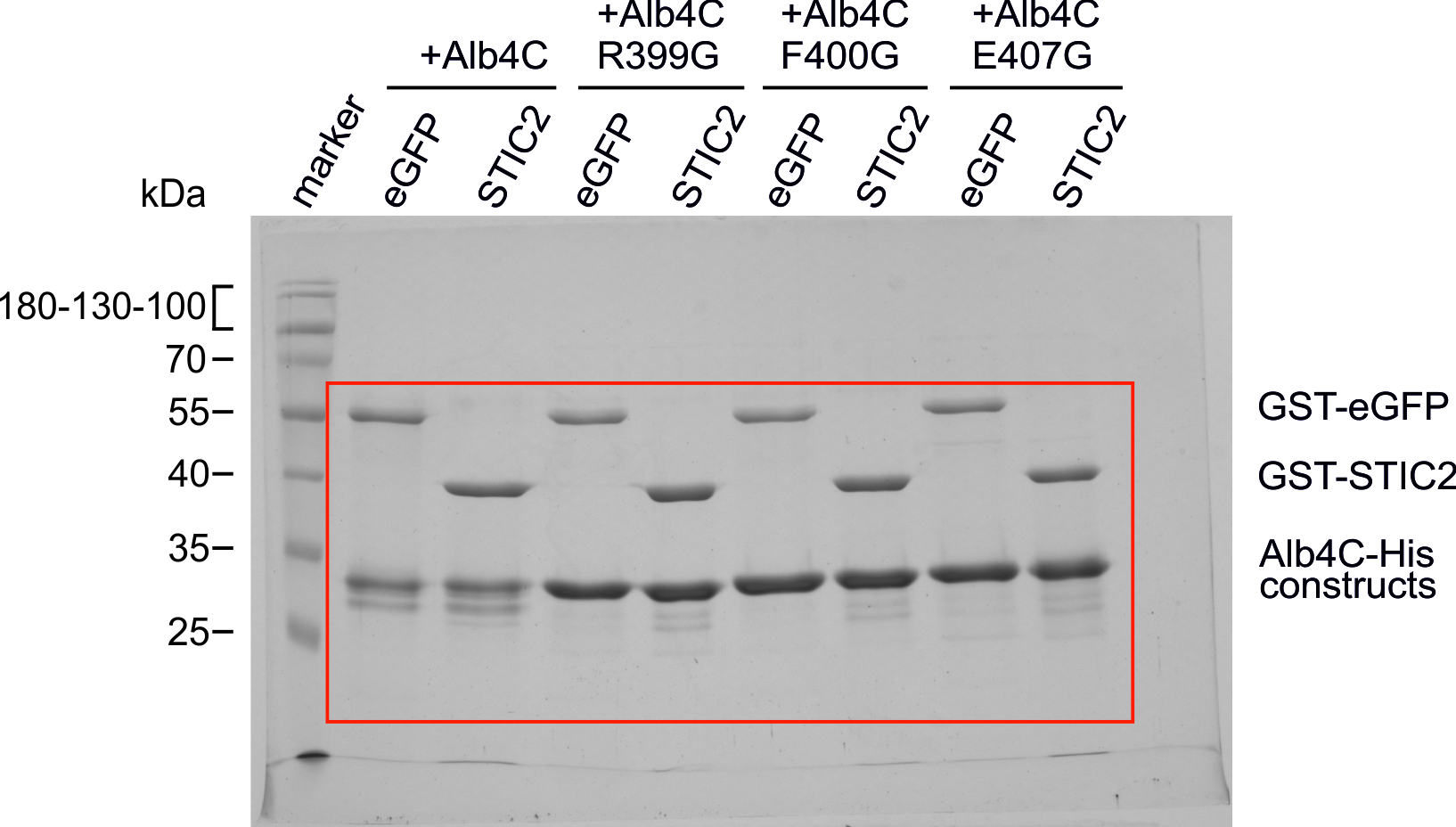

Supplement: Supplementary file 11 — Source data Fig. 6 [file 44318_2024_211_MOESM11_ESM.zip › Figure 6/6D/Coomassie left panel load.tif]

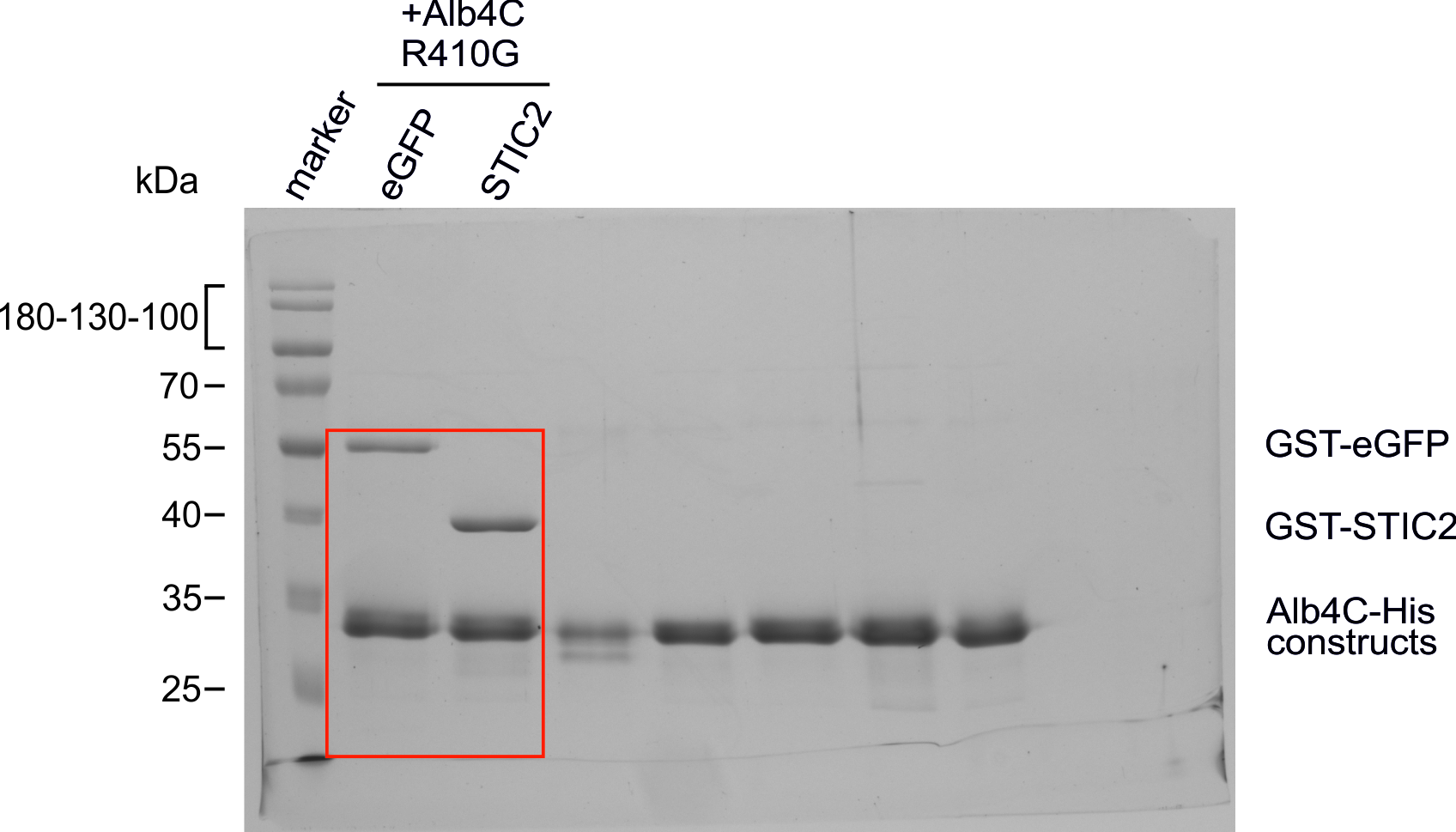

Supplement: Supplementary file 11 — Source data Fig. 6 [file 44318_2024_211_MOESM11_ESM.zip › Figure 6/6D/Coomassie right panel load.tif]

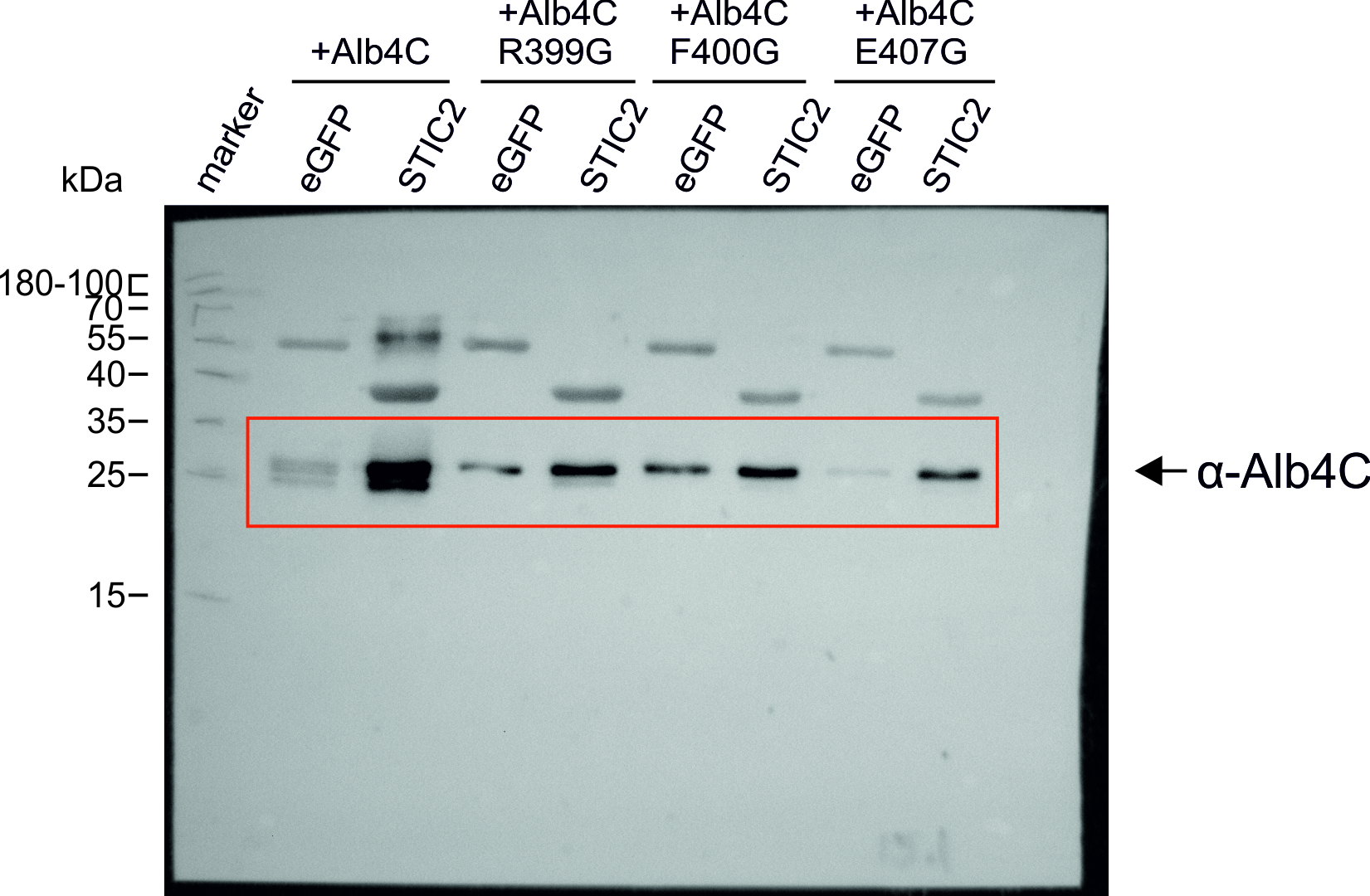

Supplement: Supplementary file 11 — Source data Fig. 6 [file 44318_2024_211_MOESM11_ESM.zip › Figure 6/6D/Western left panel Alb4C.tif]

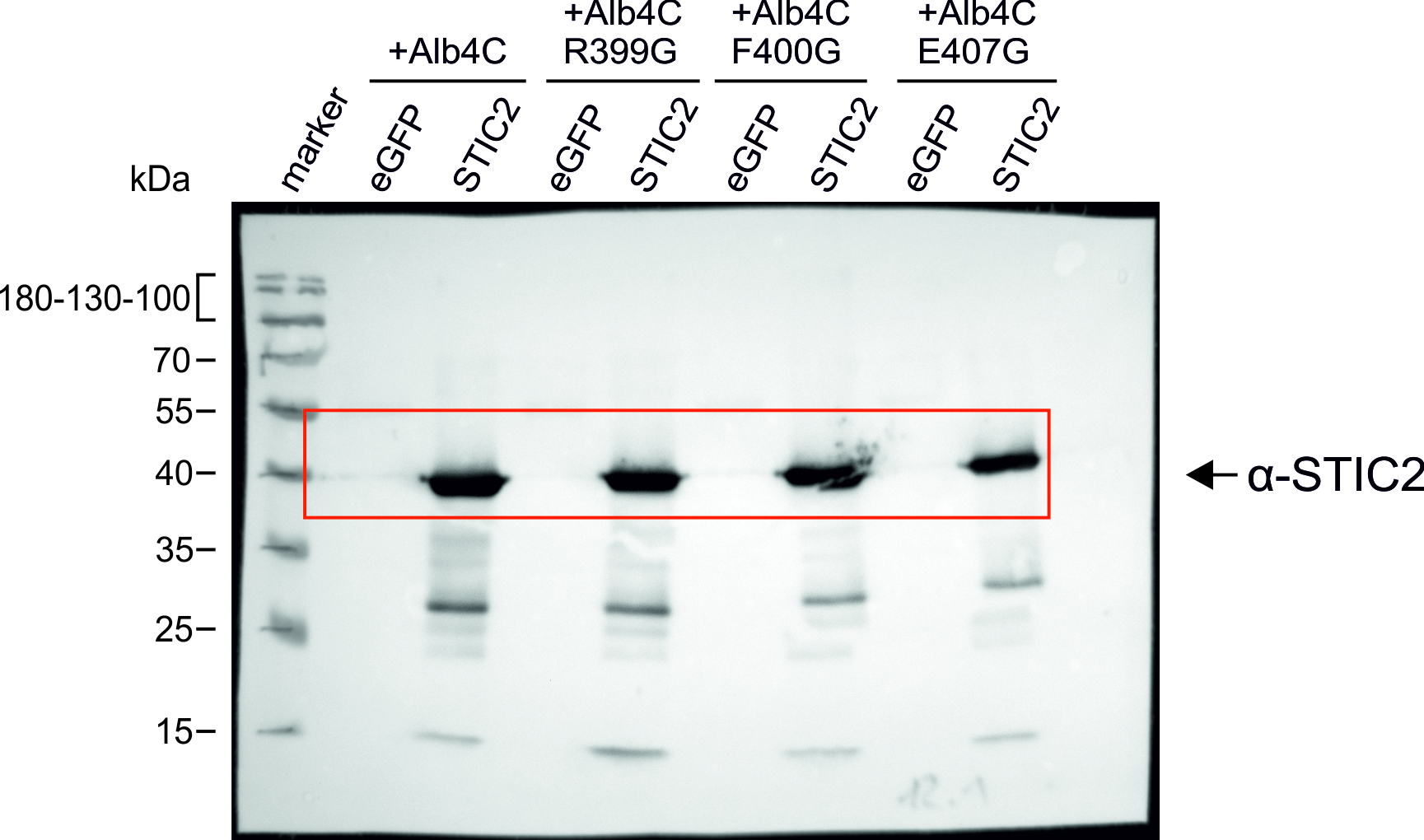

Supplement: Supplementary file 11 — Source data Fig. 6 [file 44318_2024_211_MOESM11_ESM.zip › Figure 6/6D/Western left panel STIC2.tif]

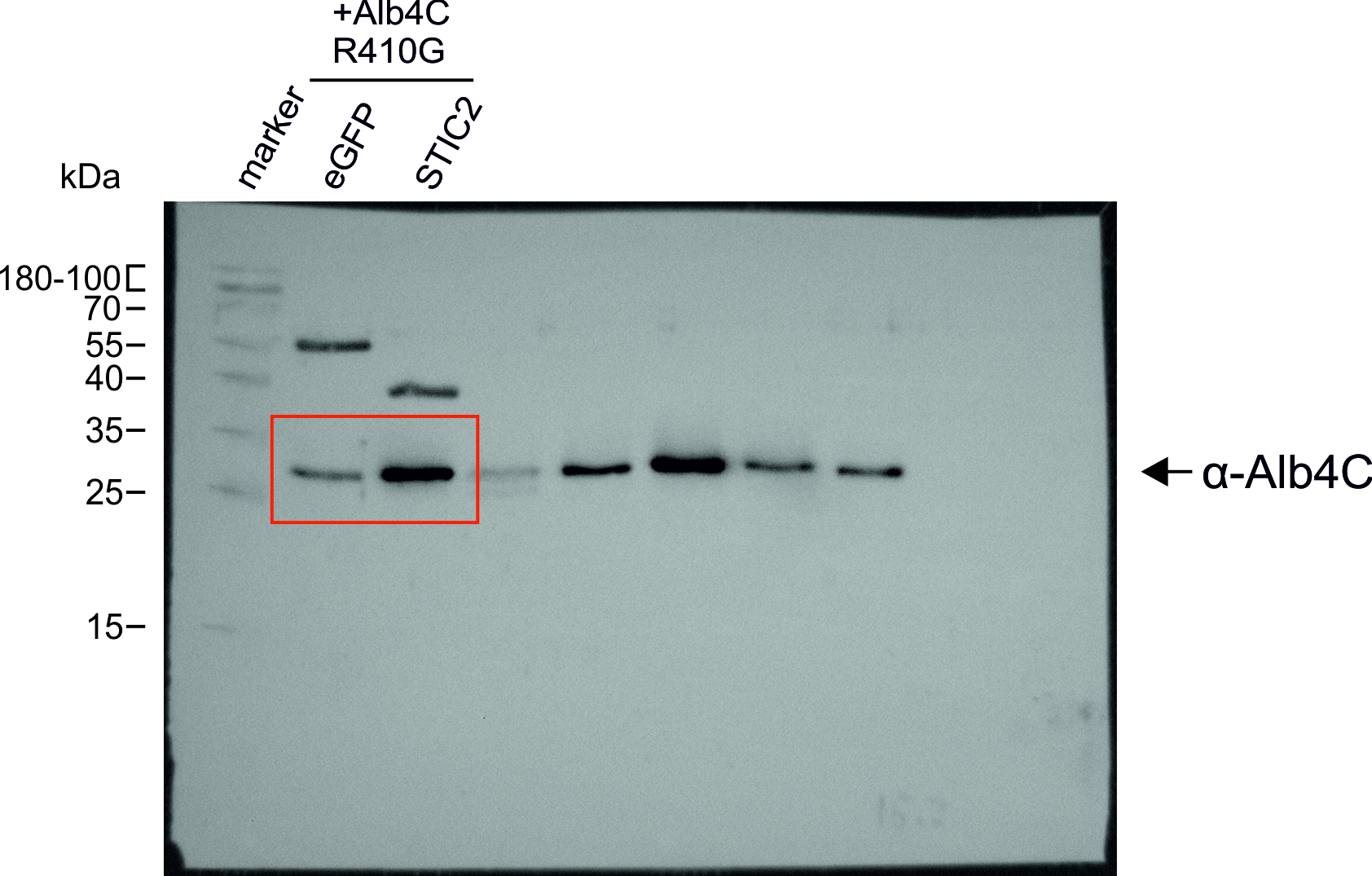

Supplement: Supplementary file 11 — Source data Fig. 6 [file 44318_2024_211_MOESM11_ESM.zip › Figure 6/6D/Western right panel Alb4C.tif]

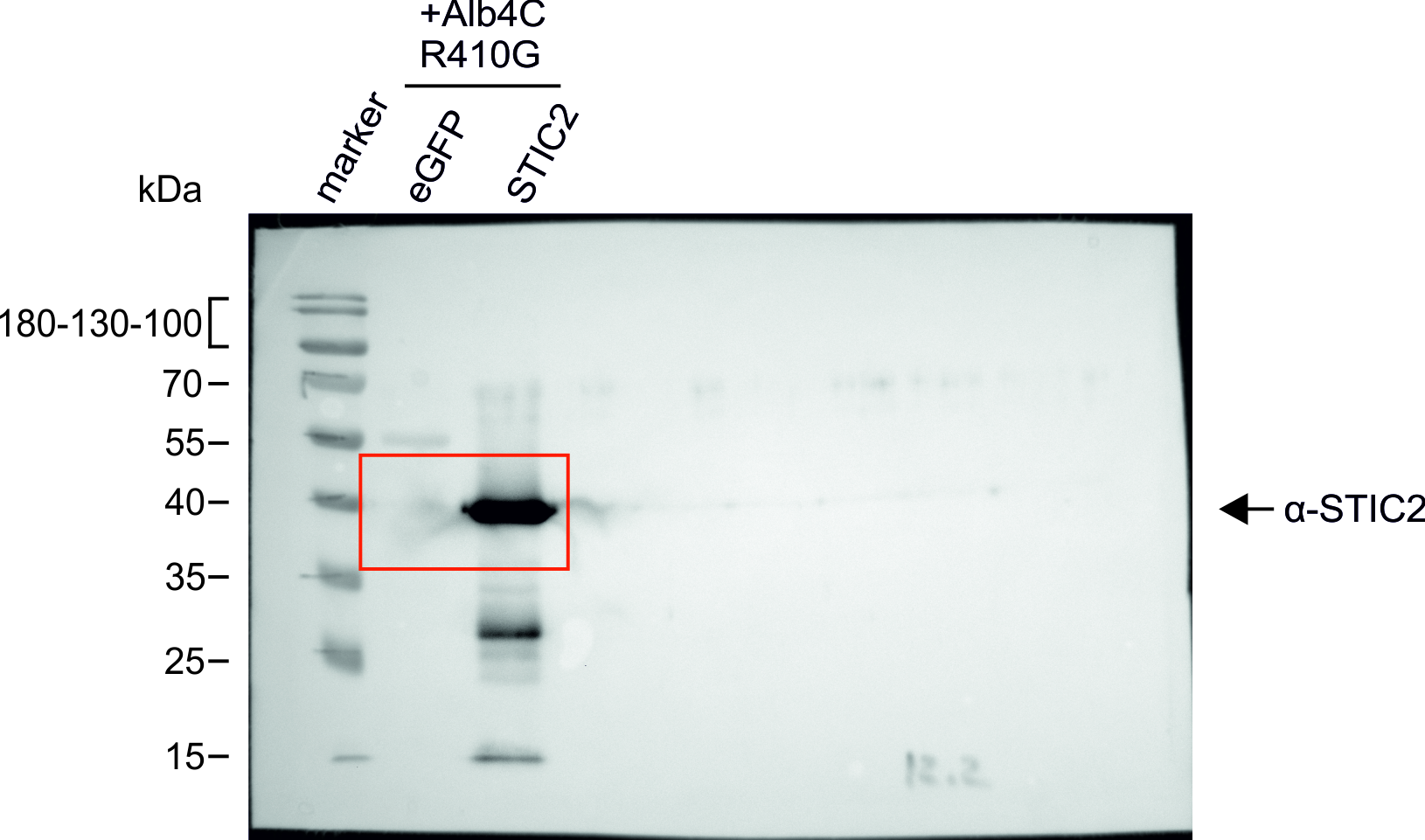

Supplement: Supplementary file 11 — Source data Fig. 6 [file 44318_2024_211_MOESM11_ESM.zip › Figure 6/6D/Western right panel STIC2.tif]

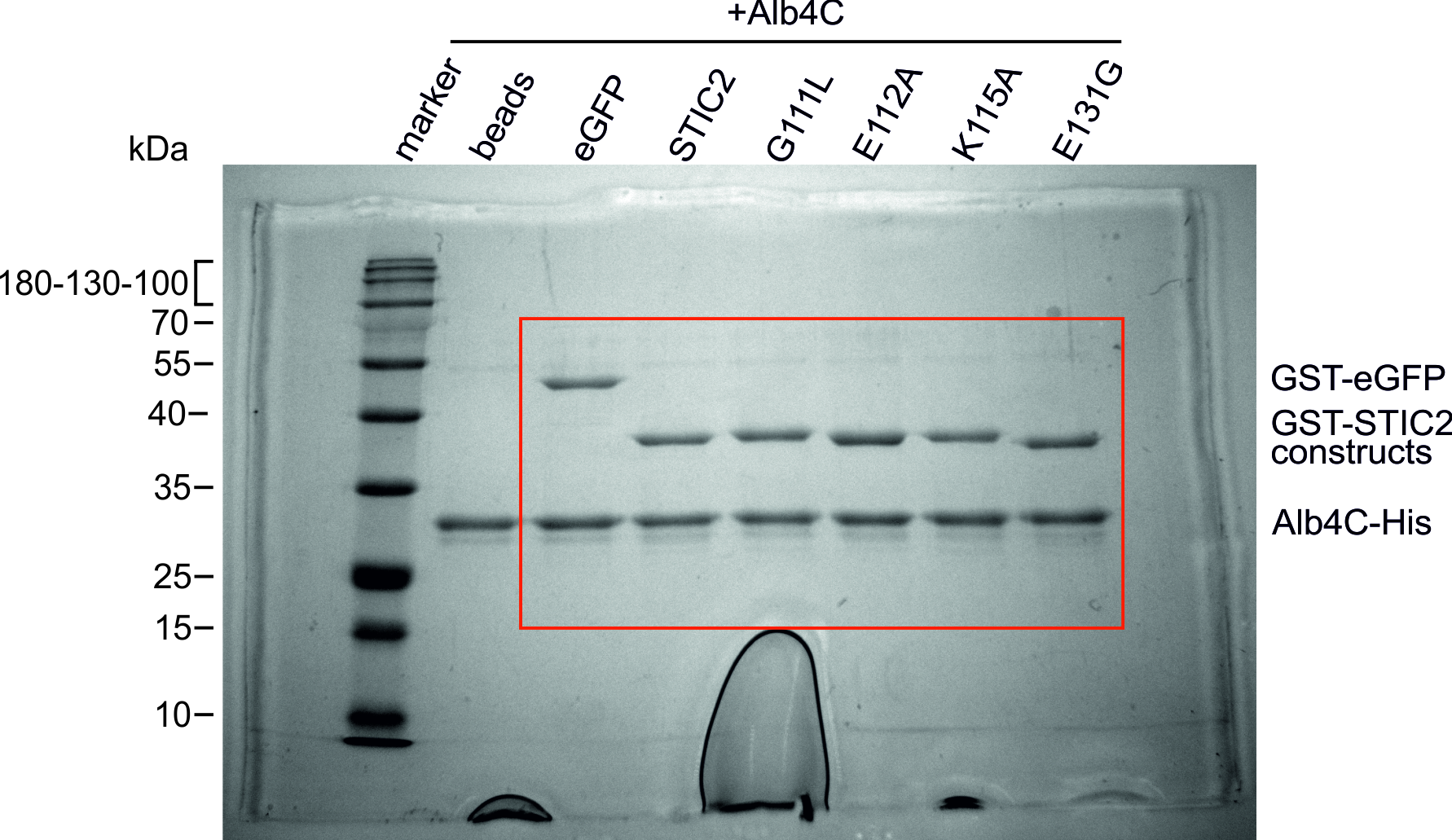

Supplement: Supplementary file 11 — Source data Fig. 6 [file 44318_2024_211_MOESM11_ESM.zip › Figure 6/6E/Coomassie left panel load.tif]

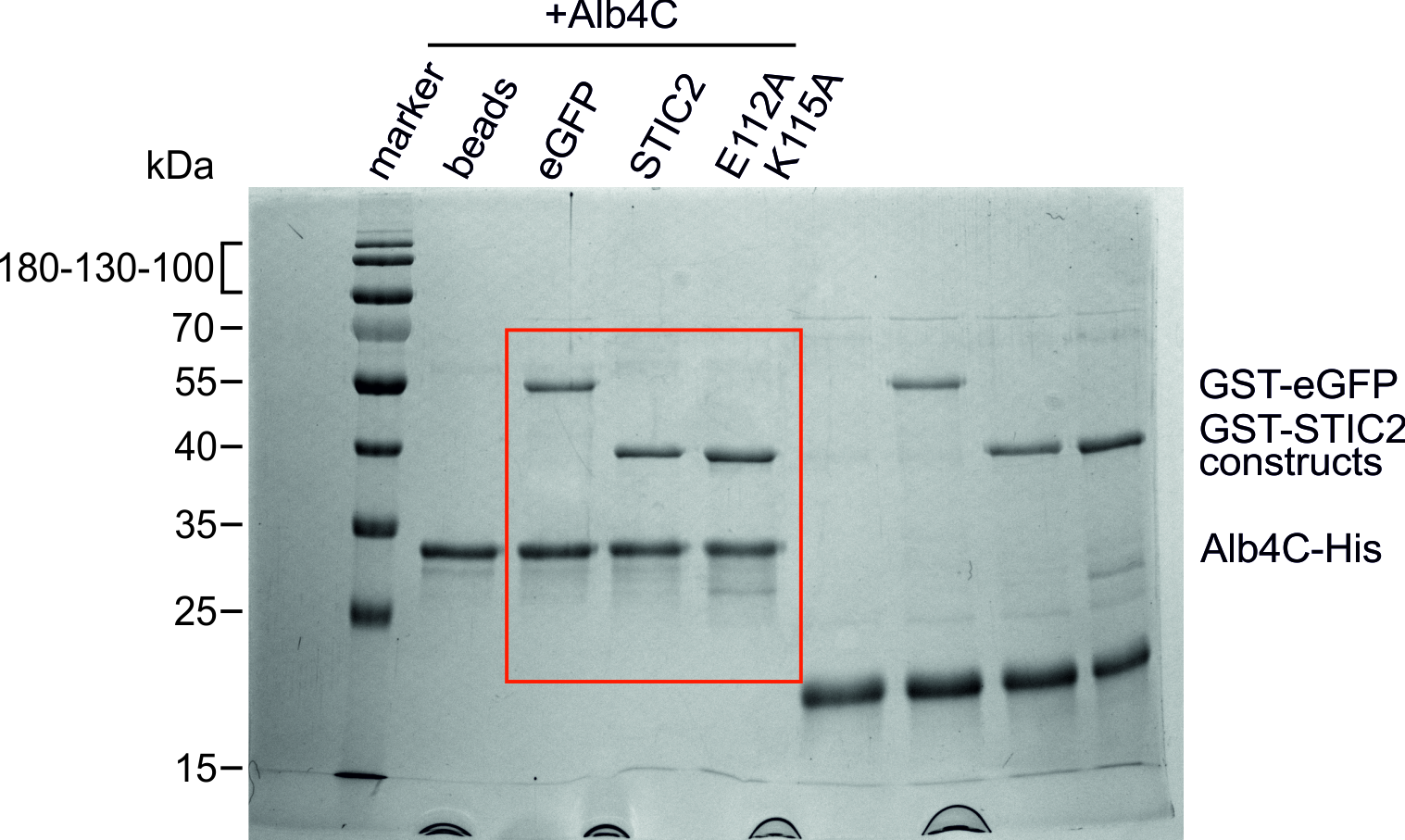

Supplement: Supplementary file 11 — Source data Fig. 6 [file 44318_2024_211_MOESM11_ESM.zip › Figure 6/6E/Coomassie right panel load.tif]

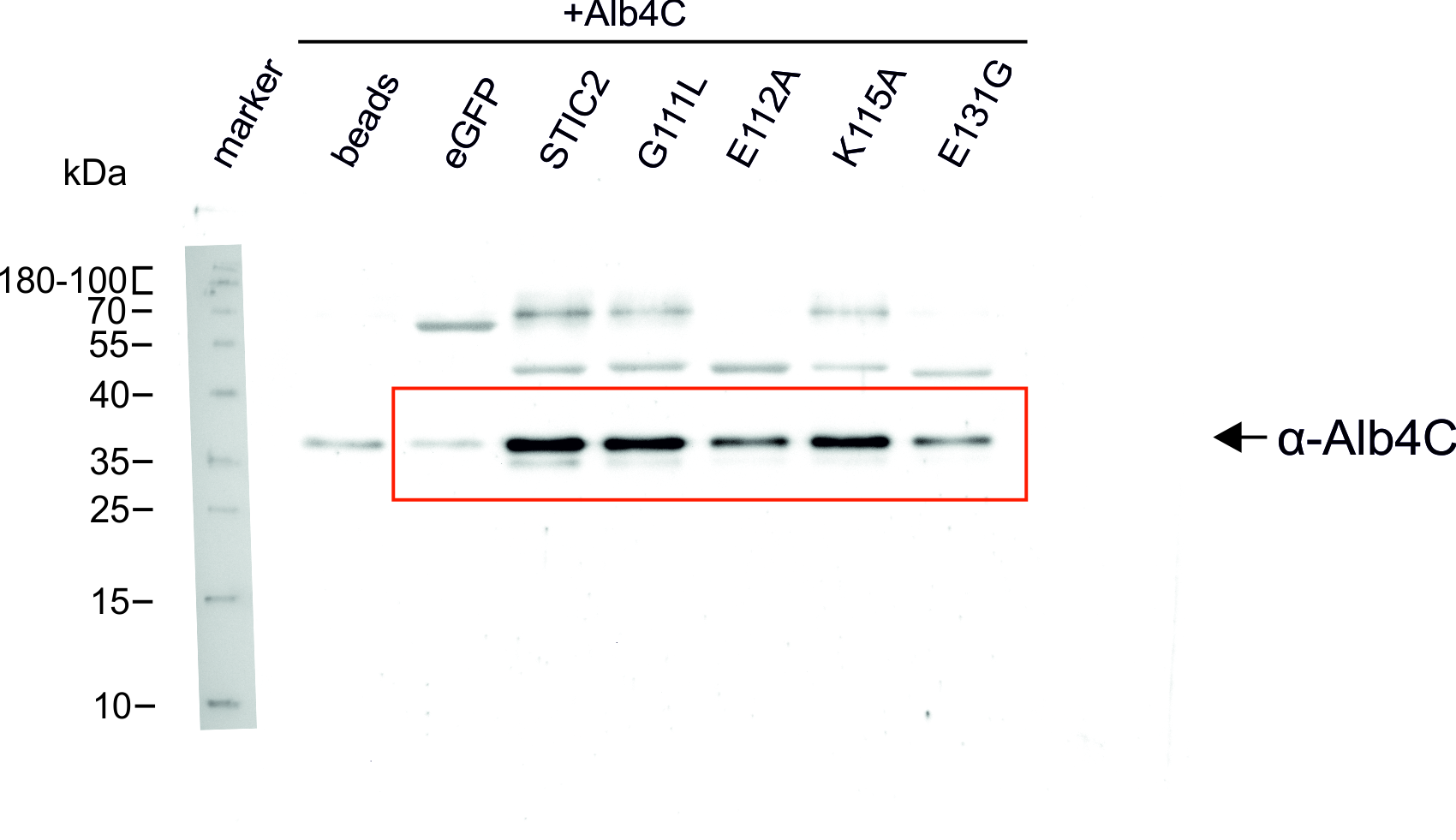

Supplement: Supplementary file 11 — Source data Fig. 6 [file 44318_2024_211_MOESM11_ESM.zip › Figure 6/6E/Western left panel Alb4C.tif]

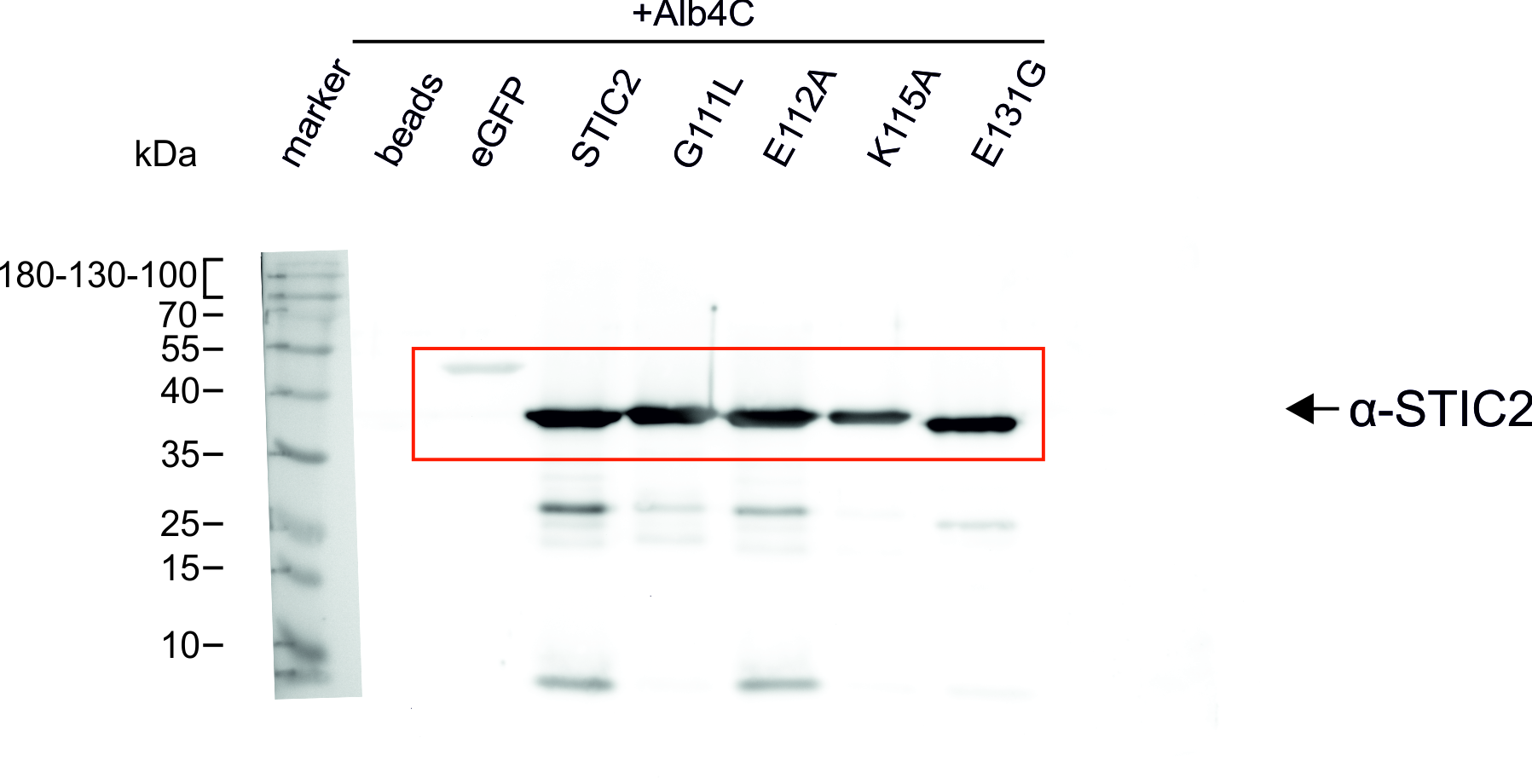

Supplement: Supplementary file 11 — Source data Fig. 6 [file 44318_2024_211_MOESM11_ESM.zip › Figure 6/6E/Western left panel STIC2.tif]

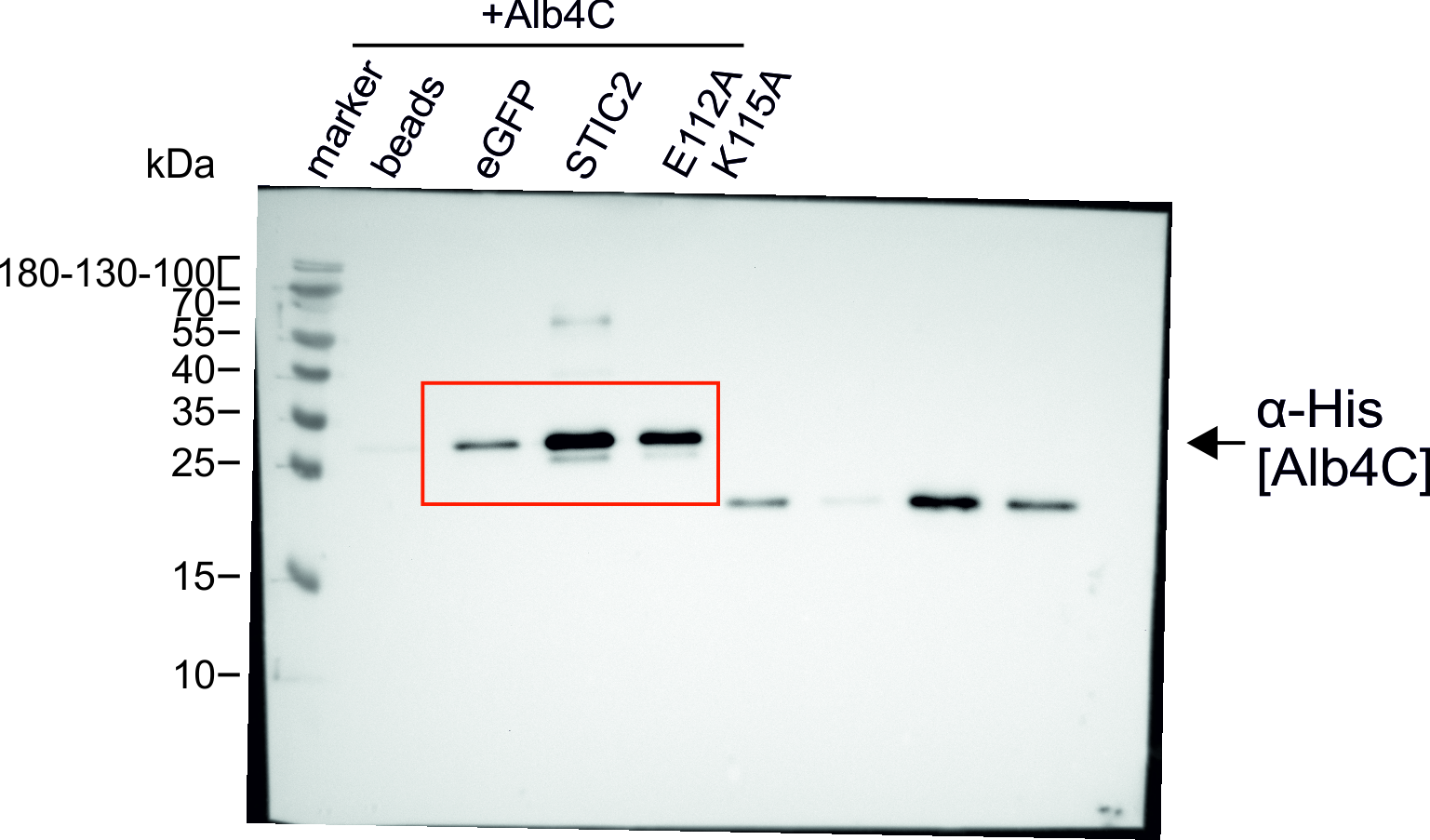

Supplement: Supplementary file 11 — Source data Fig. 6 [file 44318_2024_211_MOESM11_ESM.zip › Figure 6/6E/Western right panel His.tif]

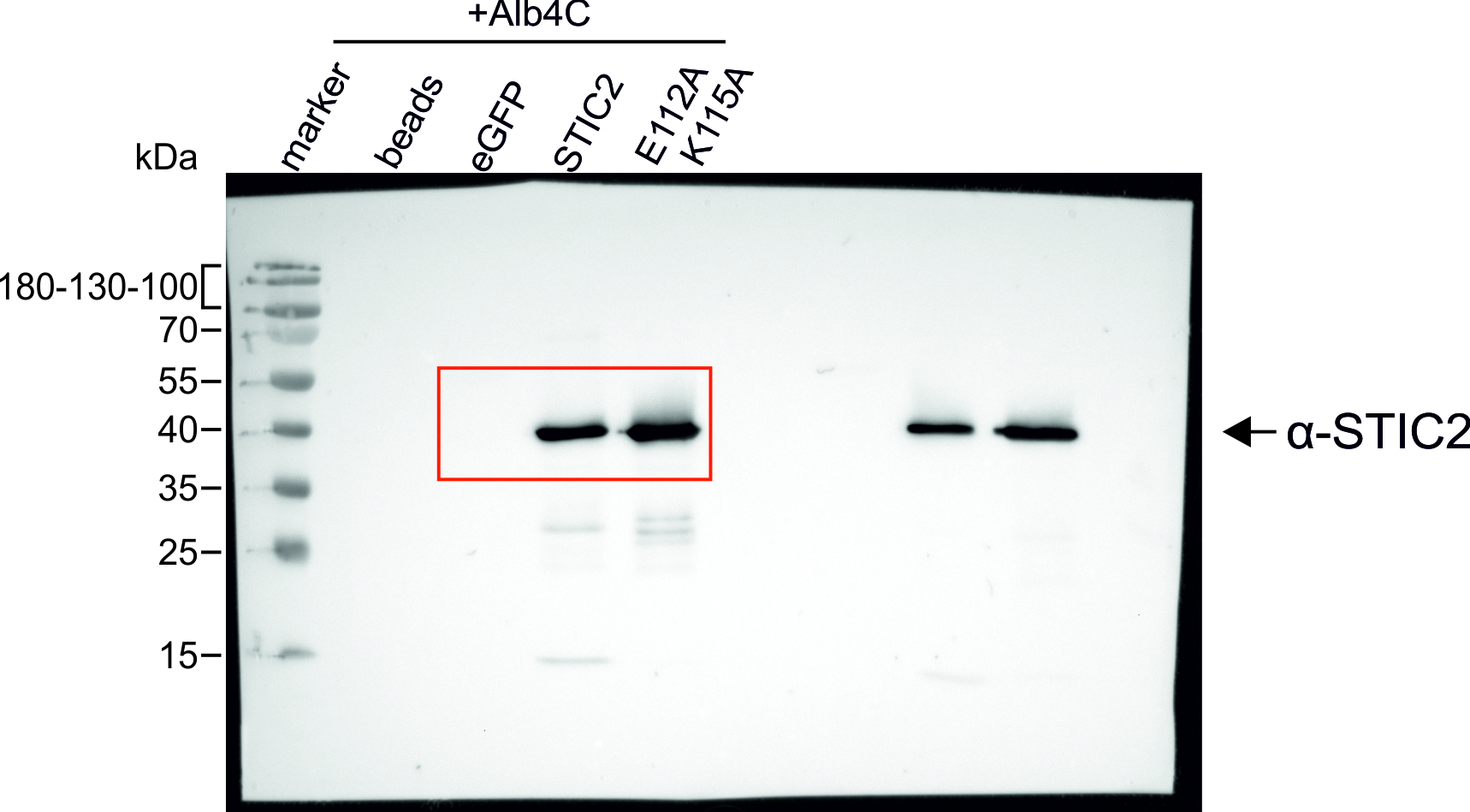

Supplement: Supplementary file 11 — Source data Fig. 6 [file 44318_2024_211_MOESM11_ESM.zip › Figure 6/6E/Western right panel STIC2.tif]

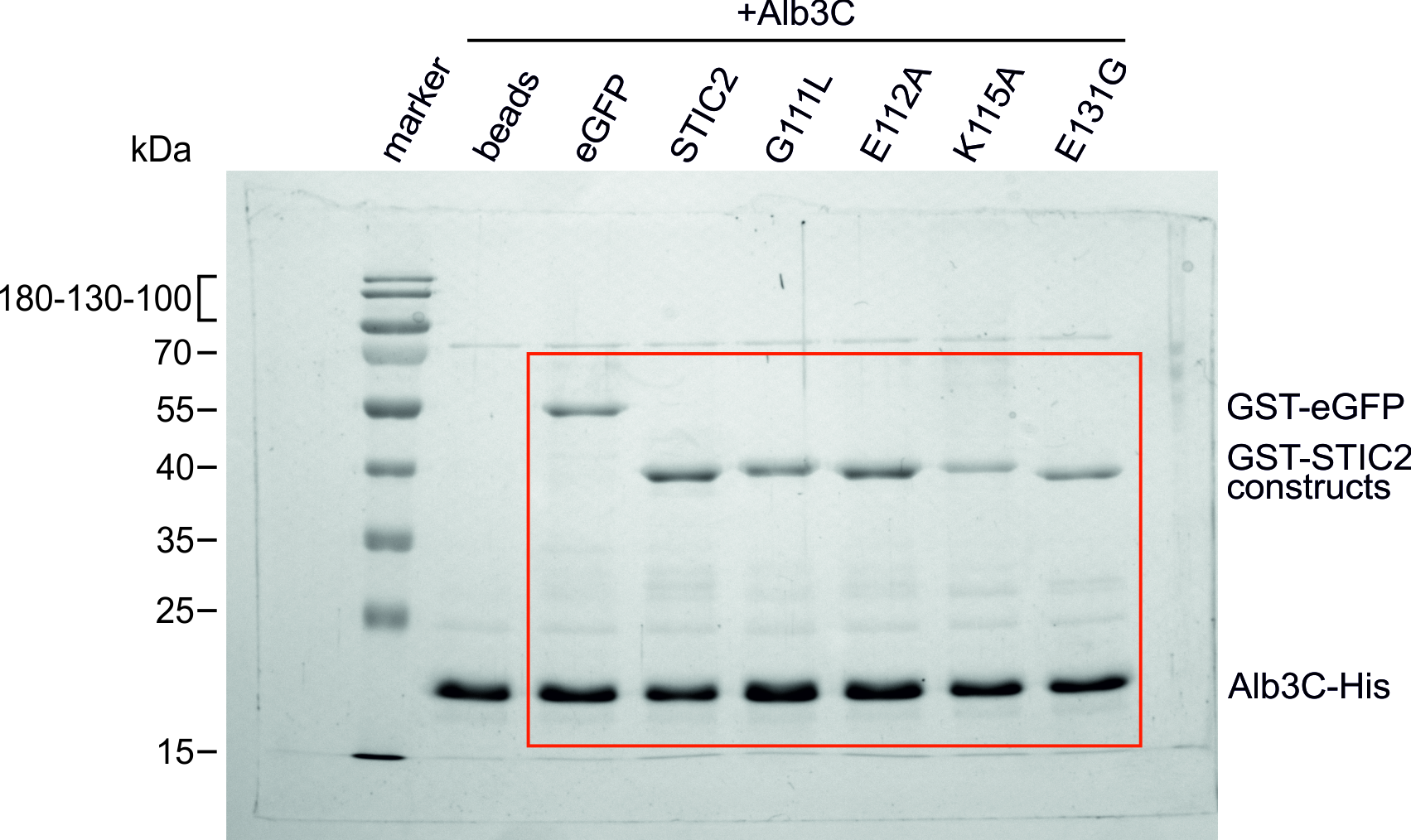

Supplement: Supplementary file 11 — Source data Fig. 6 [file 44318_2024_211_MOESM11_ESM.zip › Figure 6/6F/Coomassie left panel load.tif]

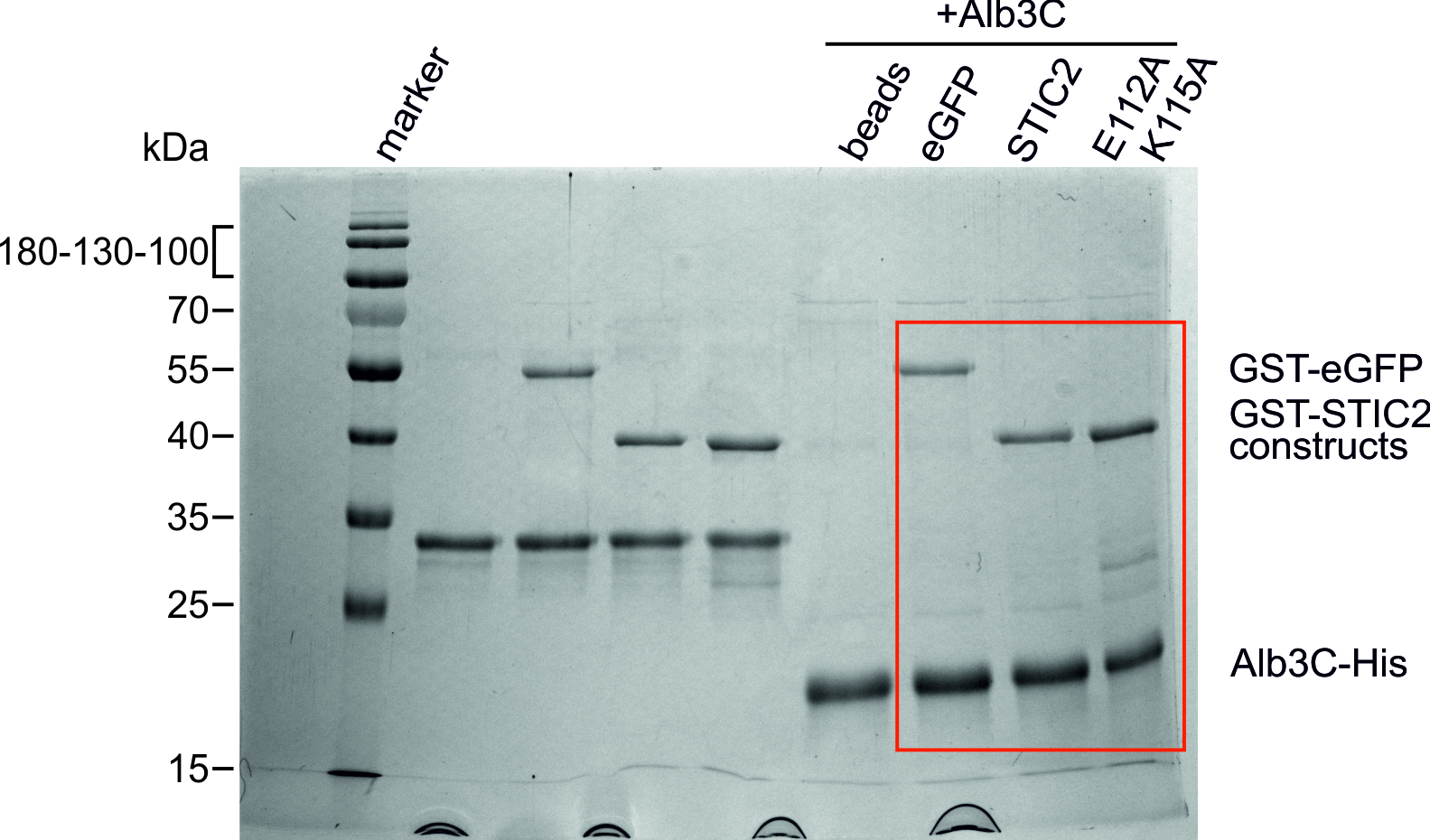

Supplement: Supplementary file 11 — Source data Fig. 6 [file 44318_2024_211_MOESM11_ESM.zip › Figure 6/6F/Coomassie right panel load.tif]

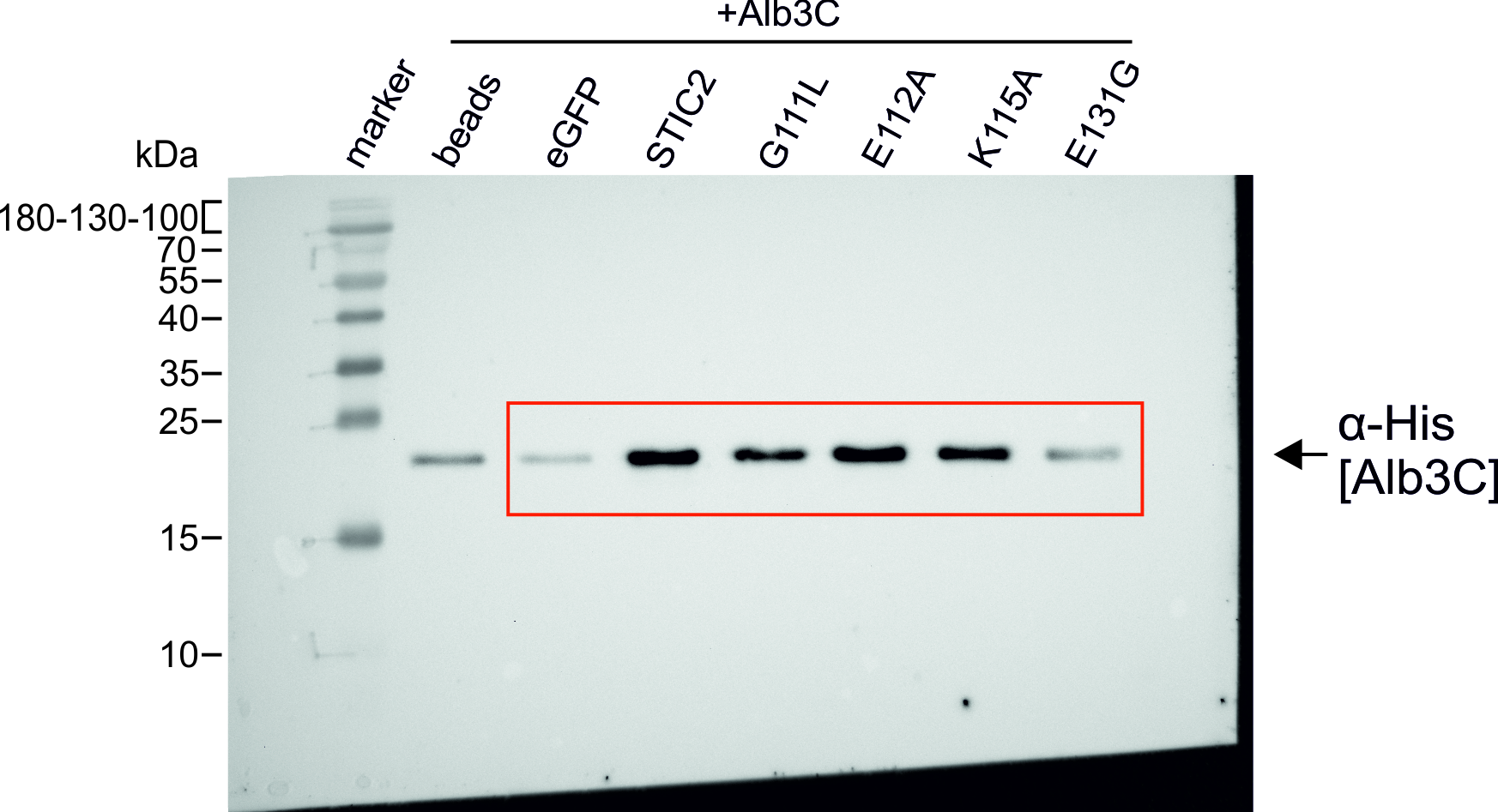

Supplement: Supplementary file 11 — Source data Fig. 6 [file 44318_2024_211_MOESM11_ESM.zip › Figure 6/6F/Western left panel His.tif]

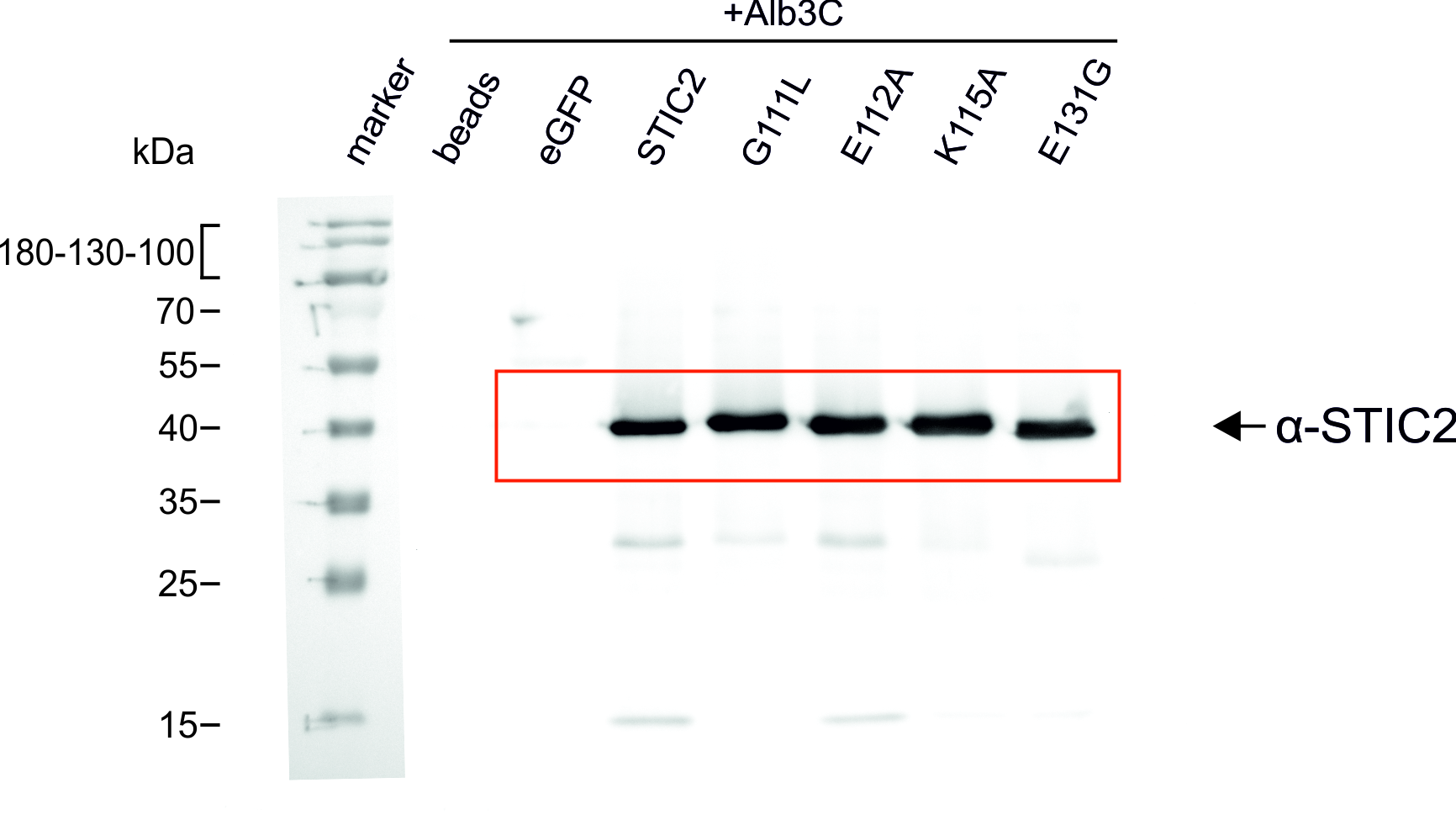

Supplement: Supplementary file 11 — Source data Fig. 6 [file 44318_2024_211_MOESM11_ESM.zip › Figure 6/6F/Western left panel STIC2.tif]

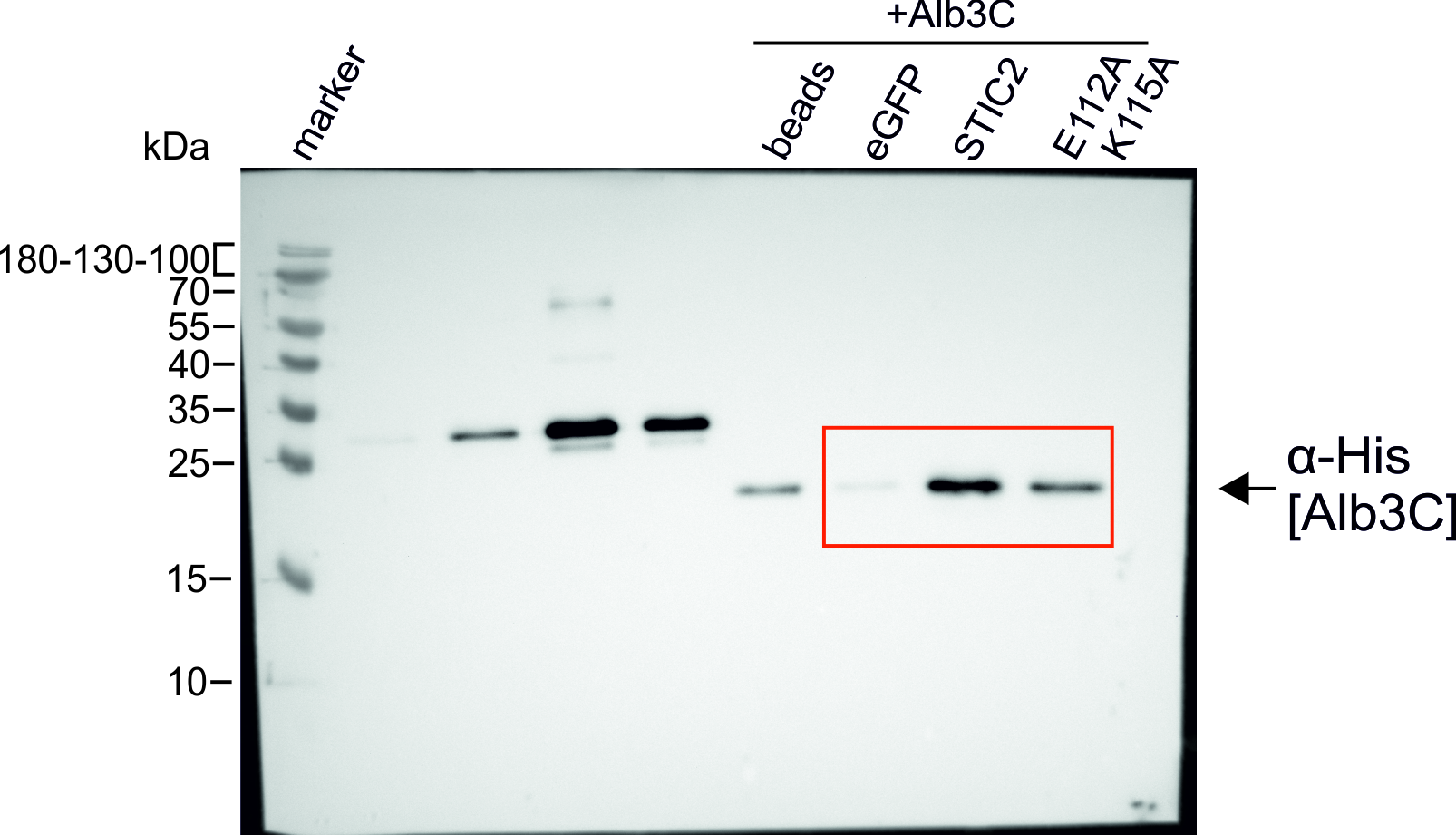

Supplement: Supplementary file 11 — Source data Fig. 6 [file 44318_2024_211_MOESM11_ESM.zip › Figure 6/6F/Western right panel His.tif]

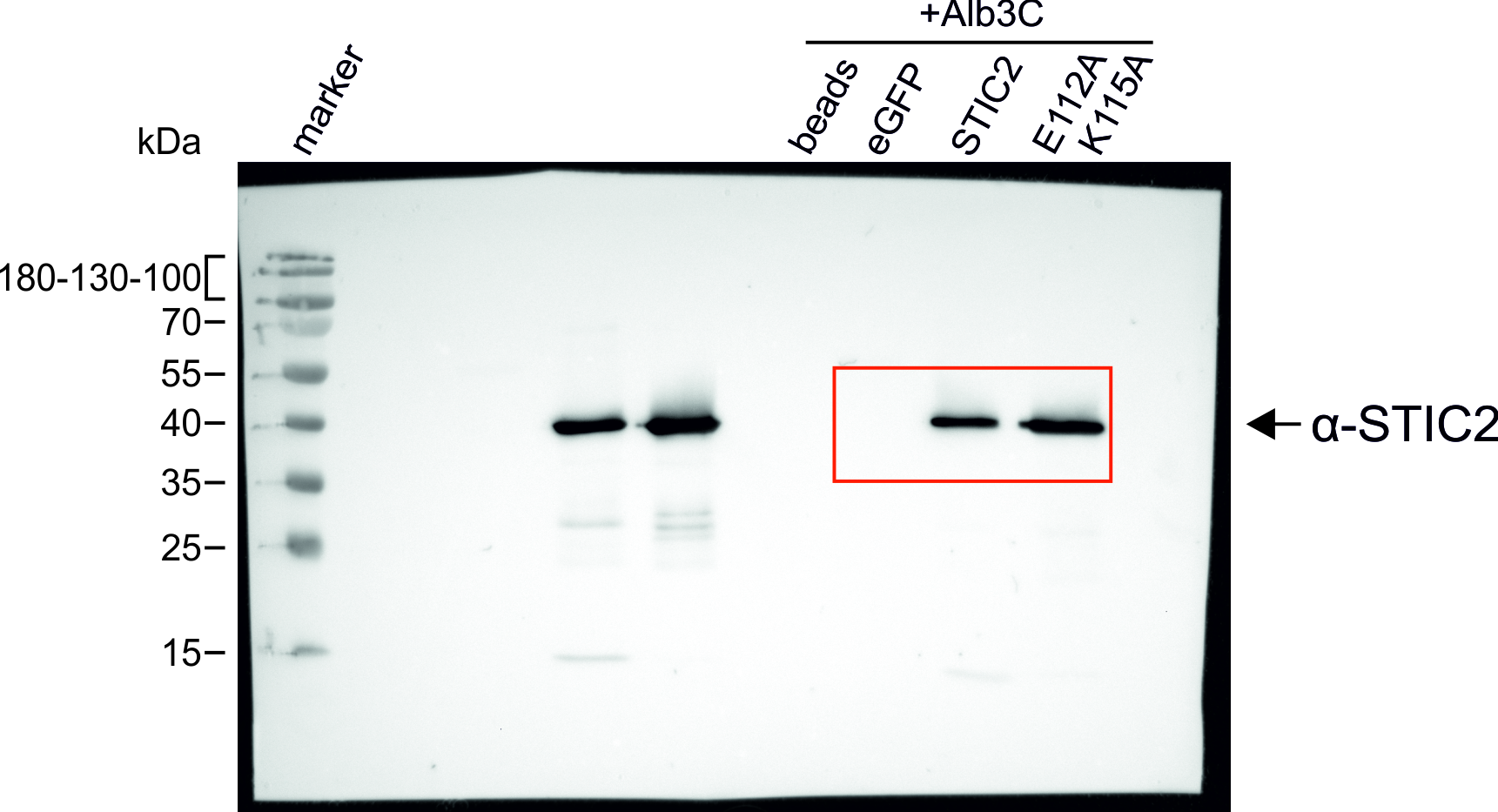

Supplement: Supplementary file 11 — Source data Fig. 6 [file 44318_2024_211_MOESM11_ESM.zip › Figure 6/6F/Western right panel STIC2.tif]
